# CD2AP deficiency aggravates Alzheimer’s disease phenotypes and pathology through p38 MAPK activation

**DOI:** 10.1186/s40035-024-00454-5

**Published:** 2024-12-19

**Authors:** Yan-Yan Xue, Zhe-Sheng Zhang, Rong-Rong Lin, Hui-Fen Huang, Ke-Qing Zhu, Dian-Fu Chen, Zhi-Ying Wu, Qing-Qing Tao

**Affiliations:** 1 Department of Neurology, The Second Affiliated Hospital, Zhejiang University School of Medicine and Liangzhu Laboratory, 88 Jiefang Road, Hangzhou, 310009 China; 2 National Health and Disease Human Brain Tissue Resource Center and Department of Pathology, School of Medicine, Zhejiang University, Hangzhou, 310058 China; 3 MOE Frontier Science Center for Brain Science and Brain-Machine Integration, School of Brain Science and Brain Medicine, Zhejiang University, Hangzhou, 310058 China; 4 CAS Center for Excellence in Brain Science and Intelligence Technology, Shanghai, 200031 China

**Keywords:** Alzheimer’s disease, CD2AP, P38 MAPK, Synaptic injury, Tau

## Abstract

**Background:**

Alzheimer’s disease (AD) is the most common form of neurodegenerative disorder, which is characterized by a decline in cognitive abilities. Genome-wide association and clinicopathological studies have demonstrated that the CD2-associated protein (*CD2AP*) gene is one of the most important genetic risk factors for AD. However, the precise mechanisms by which *CD2AP* is linked to AD pathogenesis remain unclear.

**Methods:**

The spatiotemporal expression pattern of CD2AP was determined. Then, we generated and characterized an APP/PS1 mouse model with neuron-specific *Cd2ap* deletion, using immunoblotting, immunofluorescence, enzyme-linked immunosorbent assay, electrophysiology and behavioral tests. Additionally, we established a stable *CD2AP*-knockdown SH-SY5Y cell line to further elucidate the specific molecular mechanisms by which CD2AP contributes to AD pathogenesis. Finally, the APP/PS1 mice with neuron-specific *Cd2ap* deletion were treated with an inhibitor targeting the pathway identified above to further validate our findings.

**Results:**

CD2AP is widely expressed in various regions of the mouse brain, with predominant expression in neurons and vascular endothelial cells. In APP/PS1 mice, neuronal knockout of *Cd2ap* significantly aggravated tau pathology, synaptic impairments and cognitive deficits. Mechanistically, the knockout of *Cd2ap* activated p38 mitogen-activated protein kinase (MAPK) signaling, which contributed to increased tau phosphorylation, synaptic injury, neuronal apoptosis and cognitive impairment. Furthermore, the phenotypes of neuronal *Cd2ap* knockout were ameliorated by a p38 MAPK inhibitor.

**Conclusion:**

Our study presents the first in vivo evidence that CD2AP deficiency exacerbates the phenotypes and pathology of AD through the p38 MAPK pathway, identifying CD2AP/p38 MAPK as promising therapeutic targets for AD.

**Supplementary Information:**

The online version contains supplementary material available at 10.1186/s40035-024-00454-5.

## Background

Alzheimer’s disease (AD) is the most prevalent neurodegenerative disease characterized by cognitive deficits and memory decline. Pathologically, AD is characterized by progressive synaptic loss and dysfunction, along with the accumulation of β-amyloid (Aβ) and phosphorylated tau proteins in the brain. Currently, the pathogenesis of AD remains elusive. Effective early diagnostic markers are still lacking, and no definitive curative treatments have been developed [1]. Extensive genome-wide association studies across diverse cohorts have consistently identified *CD2AP* as a genetic risk factor for AD [2–4]. A meta-analysis using large-scale samples from 15 previous studies confirmed the association of the *CD2AP* rs9349407 polymorphism with susceptibility to AD [5]. Notably, a recent two-stage genome-wide association study including 111,326 AD cases and 677,663 controls, identified rs7767350 as an AD risk locus [6]. Moreover, our previous research revealed reduced *CD2AP* expression in peripheral blood lymphocytes in sporadic AD patients, with the C allele of rs9296559 associated with an increased risk of AD [7]. Despite extensive investigations into the association between CD2AP and a greater risk of AD, the precise underlying mechanisms remain unclear [8].

The *CD2AP* gene encodes a CD2-associated protein that serves as a critical adaptor for dynamic actin remodeling and membrane trafficking [9]. The role of CD2AP in amyloidogenesis is complex and multifactorial. Notably, CD2AP has been reported to regulate Aβ generation and accumulation in neurons by increasing APP and BACE1 convergence in early endosomes [10, 11]. Intriguingly, another study reported that suppressing CD2AP expression results in decreased Aβ release and a decreased Aβ42/Aβ40 ratio in N2A cells, while global deletion of *Cd2ap* leads to a decreased Aβ42/Aβ40 ratio in the brains of 1-month-old APP/PS1 mice [12]. In addition, CD2AP plays an equally important role in tau pathology. We conducted an analysis of two distinct cohorts and revealed a correlation between *CD2AP* rs9296559 and higher CSF total tau (t-tau) and phosphorylated tau (p-tau) levels in patients with mild cognitive impairment [13]. Additionally, cindr, the fly ortholog of human CD2AP, has been identified as a modulator of tau-mediated neurotoxicity in a *Drosophila* model of AD [14, 15]. These insights indicate the potential involvement of CD2AP loss-of-function in tau-related neurodegeneration and increased susceptibility to AD. Besides, CD2AP serves as a major binding partner of endophilin and interacts with dynamin and synaptojanin, which are essential for synaptic functions [16]. CD2AP deficiency leads to compromised synaptic maturation and plasticity, underscoring the importance of CD2AP in synaptic functionality [15].

The lack of appropriate mouse models for evaluating the effects of CD2AP deficiency on learning and memory is a major challenge in the current context, primarily due to premature death from renal failure at 6–7 weeks of age in existing models [17]. In this study, we generated and characterized an APP/PS1 mouse model with neuron-specific *Cd2ap* deletion and an SH-SY5Y cell line with stable *CD2AP*-knockdown. Using these models, we comprehensively investigated AD phenotypes and pathology resulting from CD2AP deficiency both in vitro and in vivo. This study investigated Aβ and tau pathology, synaptic density and plasticity, neuronal apoptosis, and cognitive functions to elucidate the role of neuronal CD2AP in the progression of AD and the molecular mechanisms.

## Methods

### Animals

All experiments were performed in pure C57BL/6 J background. Animals were kept in our animal facility under SPF barrier, with water and food provided *ad libitum*, under controlled temperature/humidity and a 12-h dark/light cycle. All mice were housed under standard conditions with adequate water and food. Littermate mice were kept at the same cage, respecting the limit of 5 mice per cage. Mice were randomly assigned into the treatment and control groups using the drawing lots method. The order of treatments and measurements was randomized to prevent any systematic bias.

### Generation of global *Cd2ap* knockout mice and neuronal *Cd2ap* knockout mice

*Cd2ap*^fl/fl^ mice were generated by Biocytogen (Beijing, China) using the CRISPR/Cas9 method. In Syn1-iCre mice (B-CM-005, Beijing Biocytogen Co., Ltd), an F2A-iCre sequence cassette was placed between the exon 13 and 3’UTR of the *Syn1* gene using CRISPR/Cas9 technology. The *Cd2ap*^fl/fl^ mice were crossed with CMV-Cre and Syn1-iCre mice to generate global *Cd2ap* knockout and conditional neuronal *Cd2ap* knockout mice, respectively. The *Cd2ap*^fl/fl^ mice were crossed with CMV-Cre mice to generate *Cd2ap*^+/-^ mice, which were then bred to produce global *Cd2ap* knockout mice. For the neuronal *Cd2ap* knockout mice, female *Cd2ap*^fl/fl^ Syn1-iCre mice were mated with male *Cd2ap*^fl/fl^ mice. Genotyping was performed by polymerase chain reaction (PCR) using DNA isolated from mouse tails. The PCR primers used for mouse genotyping are listed in **Table S1**.

### Behavioral analyses

#### Open field test

The open field test was performed to examine exploration-related motivation and anxiety-like behavior. Mice were introduced to the behavioral experimental room at least 30 min before the behavioral trials. At the start of each trial, the mice were transferred to the center of the chamber (40 cm × 40 cm × 40 cm) and allowed to freely explore the environment for 5 or 10 min. Time spent in the central area was calculated. The surface of the chamber was cleaned with 70% ethanol and thoroughly dried between trials.

#### Elevated plus maze test

The elevated plus maze test was carried out to assess anxiety-related responses. Mice were placed in the center of the apparatus, which consisted of four elevated arms (each arm measuring 30 cm × 5 cm, with 25-cm-tall walls on the two closed arms). Mice were allowed to freely explore the apparatus for 5 min, and the time spent in the open arms was analyzed.

#### Novel object recognition test

Novel object recognition test was performed to assess object recognition memory. Twenty-four hours prior to the test, all mice were consecutively habituated to an empty chamber for 10 min. The experiments were carried out in two stages. During the first stage, the mice were returned to the same chamber and exposed to two identical objects for 5 min. Two hours later, one object was replaced with a novel object, and the mice were then returned to the chamber for another 5 min. The movements of the mice were tracked and recorded by an overhead video camera. The interaction time with the objects was analyzed by an observer blinded to the genotype. The discrimination index was then calculated as follows:

$$\text{Discrimination index = }\frac{\text{Time }\left({\text{novel}}\right) \text{- Time (familiar)}}{\text{Time }\left({\text{novel}}\right)\text{ + Time (familiar)}}$$

#### Morris water maze

Morris water maze test was performed to evaluate hippocampus-dependent spatial memory. The maze was a circular tank (100 cm in diameter) filled with opaque water, with visible cues of different geometrical symbols attached to the walls. During the training days, a mouse was placed in the maze to locate a hidden platform submerged approximately 1 cm below the water surface (60 s per trial, 4 trials per day, with 30-min intervals between trials). Once the platform was reached, the mouse was given a 10-s rest. If the mouse failed to find the platform within the time limit, it was manually guided to the platform. After consecutive 4 days of trial, the platform was removed, and mice were allowed to swim freely for 60 s. Tracks were recorded and analyzed with a camera connected to WaterMaze TM software version 4.07 (ActiMetrics, Inc., Lafayette, IN).

#### Y-maze novel arm preference test

This test was performed to assess spatial and working memory in mice. The Y-shaped maze consisted of three arms (A, B, and C arms) of equal length (35 cm), arm lane width (10 cm), and wall height (25 cm). The test was divided into two stages. In the first stage, a removable obstacle was placed to block access to the C arm. Mice were placed in arm A and allowed to explore arms A and B freely for 10 min. After one hour, the obstacle was removed in the second stage. Mice were returned to arm A and allowed to explore the three arms freely for 5 min. The time spent in arm C was analyzed and compared among the groups.

#### Fear conditioning test

An apparatus consisting of two chambers with distinct contexts was used for the measurement of contextual and cued fear conditioning tests. On the first day, a mouse was placed in a conditioning box with a grid floor. The mouse was allowed to explore the box freely for 2 min and then received a foot shock (0.6 mA) during the last 2 s of a 30-s tone (80 dB). This tone-foot shock cycle was repeated three times with an interval of 30 s, and the mouse was allowed to explore the box freely for 90 s before returning to its home cage. The next day, the mouse was placed in the original conditioning box and allowed to explore freely for five minutes. For the auditory-cued freezing test, the mouse was placed in a box different from the original box. The mouse was allowed to explore the box freely for three minutes, and a tone (80 dB) sounded for 3 min, after which it was allowed to explore freely for 90 s. During the tests, freezing values were recorded automatically by the software package Packwin (Mobile Datum, Shanghai, China).

### Drug administration

SB203580 (Selleck Chemicals, Shanghai, China) was dissolved in 2% DMSO, 30% PEG400 and 5% Tween 80, and administered intraperitoneally at a dose of 0.5 mg/kg. The dose and route of SB203580 administration were determined based upon previous studies with minor modifications [18, 19].

### Tissue collection

The mice were deeply anesthetized with isoflurane and transcardially perfused with 0.1 mol/l PBS (pH 7.4). The brains were quickly removed and hemisected. The left hemisphere was frozen in liquid nitrogen, and the right hemisphere was kept in 4% (*w*/*v*) paraformaldehyde for 24 h at 4 °C. After dehydration in 30% (*w*/*v*) sucrose, the brains were embedded in optimal cutting temperature (OCT) compound and cut into 12-μm-thick sections on a cryostat (LEICA, Wetzlar, Germany).

### Immunofluorescence analysis

The brain sections were blocked in blocking buffer (5% donkey serum, 1% BSA, and 0.3% Triton X-100) for 2 h at 4 °C and then incubated overnight at 4 °C with the following primary antibodies: anti-CD2AP (1:200, Sigma-Aldrich, St. Louis, MO, HPA003326), anti-CD31 (1:300, R&D Systems, Minneapolis, MN, AF3628), anti-NeuN (1:200, Sigma, MAB377), anti-NeuN (1:200, Abcam, Cambridge, England, ab104224), anti-GFAP (1:500, Millipore, Billerica, MA, MAB360), anti-IBA1 (1:300, Abcam, ab5076), anti-β-amyloid (1:400, BioLegend, San Diego, CA, 803,001), anti-Aβ42 (1:100, Sigma-Aldrich, AB5078P), anti-amyloid fibrils OC (1:200, Sigma-Aldrich, AB2286), anti-β-amyloid, 17–24 antibody (clone 4G8) (1:100, Biolegend, 800701) and anti-synaptophysin (1:500, Sigma-Aldrich, S5768). After being washed with PBS, the brain sections were incubated for two hours at room temperature with the corresponding secondary antibodies (1:500, Thermo Fisher Scientific, Waltham, MA). The cell nuclei were then counterstained with 4,6-diamino-2-phenylindole (DAPI) for 15 min at room temperature, after which the brain sections were coverslipped with an antifading liquid mountant.

### Gallyas silver staining

A modified Gallyas silver staining method was used to detect the neurofibrillary tangles [20]. The paraffin sections were cut at a thickness of 15 μm, mounted on positively charged microscope slides, and allowed to dry overnight before staining. After deparaffinization with xylene and hydration with gradient ethanolic concentrations (100% to 70%), the slides were washed in distilled water for 5 min and transferred immediately to 5% periodic acid for 30 min. Subsequently, the slides were washed twice for 5 min in distilled water before being incubated in silver iodide solution (4 g sodium hydroxide, 10 g potassium iodide, 0.7 ml of 5% silver nitrate in 100 ml distilled water) for 30 min. Following three rinses (10 s each) in distilled water, the slides were washed in 0.5% acetic acid for 5 min. The slides were then incubated in physical developer solution (solution A: 50 g of sodium carbonate in 1000 ml of distilled water; solution B: 2 g of ammonium nitrate, 2 g of silver nitrate, and 10 g of tungstosilicic acid in 1000 ml of distilled water; and solution C: 2 g of ammonium nitrate, 2 g of silver nitrate, 10 g of tungstosilicic acid, and 7.5 ml of 35% formaldehyde in 1000 ml of distilled water) for 30 min. The developer solution was prepared fresh before use at a 10:3:7 ratio (solution A:solution B:solution C). Next, the slides were washed in 0.5% acetic acid for 5 min, followed by two washes with distilled water for 5 min each. After that, the slides were fixed with 5% sodium thiosulfate for 15 min. Then, the slides were washed in distilled water twice (5 min each), dehydrated in alcohol, cleared with xylene, and finally mounted using Permount solution.

### Cell culture and plasmid transfection

SH-SY5Y cells were cultured in DMEM supplemented with 10% fetal bovine serum (Gibco, Logan, UT) in 5% CO_2_ at 37 °C. SH-SY5Y cells were transfected with APPSwe or empty vector plasmid using Lipofectamine® 3000 reagent (Invitrogen, Carlsbad, CA) according to the manufacturer’s protocol. Twenty-four or forty-eight hours after transfection, the transfected cells were collected for further analysis.

### Lentiviral infection

Stable *CD2AP* knockdown was achieved by lentiviral infection. SH-SY5Y cells were infected with control lentiviral particles (LV Sh-Ctrl) or CD2AP/GFP shRNA (LV Sh-CD2AP, Neuron Biotech, Shanghai, China). The control shRNA was generated in the pLKD.CMV.GFP.U6.shRNA vector with the target sequence 5'- TTCTCCGAACGTGTCACGT-3'. The target sequences of the CD2AP shRNAs were as follows: ShRNA1, 5'-GCCAGTAATTTACTGAGATCT-3'; ShRNA2, 5'-GGAGCTGAAAGTGGGAGATAT-3'; ShRNA3, 5'-GGACTTCCAGCTGGAGGAATT-3'. After lentiviral transduction, target SH-SY5Y cells were selected using 1.2 mg/ml geneticin (Beyotime, Shanghai, China, ST081) for 14 days. The stably infected SH-SY5Y cells were then cultured in 0.6 mg/ml geneticin and used directly in subsequent studies.

### RNA isolation and quantification

Total RNA was extracted from human and mouse tissues using TRIzol reagent, and 0.5 μg of RNA was reverse transcribed to cDNA using PrimeScript™ RT Master Mix (TaKaRa, Kusatsu, Japan, RR036A). Quantitative real-time PCR experiments were further performed using TB Green Premix Ex Taq (TaKaRa, RR420A). GAPDH was used as an internal control, and the relative gene expression was calculated using the 2^−ΔΔCt^ method. The primer sequences are provided in **Table S1**.

### Western blot analysis

Tissues or cells were lysed in RIPA lysis buffer containing a protease and phosphatase inhibitor cocktail (Roche, Basel, Switzerland) and 1 mM PMSF. Following centrifugation at 12,000 × *g* for 20 min at 4 °C, protein samples were collected and quantified using a BCA protein assay kit (TaKaRa). After boiling with loading buffer for 10 min, protein samples were separated by 8%, 10%, or 12% SDS‒PAGE and then transferred to a PVDF membrane (Millipore) by electrophoresis. After being blocked in 5% fat-free milk for one hour at room temperature, the membranes were incubated separately with primary antibodies overnight at 4 °C, followed by incubation with the corresponding HRP-conjugated secondary antibodies for one hour at room temperature. Primary antibodies against the following targets were used: CD2AP (1:1000, Sigma-Aldrich, HPA003326), phosphor-p38 MAPK (1:1000, Cell Signaling Technology, Danvers, MA, 4511S), p38 MAPK (1:1000, Cell Signaling Technology, 8690S), p-tau396 (1:1000, Invitrogen, 44-752G), AT8 (1:500, Invitrogen, MN1020), tau-5 (1:500, Invitrogen, AHB0042), synaptophysin (1:1000, Sigma-Aldrich, S5768), caspase-3 (1:1000, Proteintech, Rosemont, IL, 66470–2-Ig), cleaved caspase-3 (1:1000, Cell Signaling Technology, 9664S), APP-Y188 (1:1000, Abcam, ab32136), cyclin kinase-5 (CDK5) (1:1000, Abclonal, A5730), p35/p25 (1:1000, Cell Signaling Technology, 2680S), GSK3β (1:1000, Abclonal, Wuhan, China, A11731), phospho-GSK3β-S9 (1:1000, Abclonal, AP0039), GAPDH (1:20,000, Abclonal, AC035), and β-actin (1:10,000, Abclonal, AC043). The gray values of the bands were analyzed by FIJI-ImageJ software (NIH, Bethesda, MD).

### Quantitative immunofluorescence analysis

For colocalization analysis, Mander’s overlap coefficients were assessed using the Coloc2 plugin in FIJI-ImageJ software. The mean fluorescence intensity of CD2AP within the boundaries of the different cellular markers was determined using the ROI Manager tool in FIJI. In addition, the FIJI-ImageJ software was used to measure the synaptophysin density in the DG region, CA3 region, entire hippocampus, and cortex. Brain slices of similar hippocampal sizes were selected for immunofluorescence staining, photographed at 20 × magnification, and then stitched together to create a composite image. The quantification analysis was conducted by a researcher who was blinded to the genotypes. Multiple images were selected within each section to cover the DG region, CA3 region, and cortex. The threshold was equally adjusted across all brains to highlight the stained area, and particles were analyzed to determine the total area stained per total area analyzed.

### Enzyme-linked immunosorbent assay (ELISA)

Cortical tissues were dissected and sonicated in cold RIPA lysis buffer, followed by centrifugation at 12,000 × *g* for 20 min at 4 °C. The supernatant was collected for measurement of RIPA-soluble Aβ protein level. The deposits were further processed by an ultrasonic homogenizer with 100 μl of 70% formic acid in ddH_2_O. After centrifugation, the supernatant was collected and neutralized with 1 M Tris buffer for further measurement of the concentration of the RIPA-insoluble Aβ protein. RIPA-soluble and formic acid-soluble Aβ40 and Aβ42 were assayed by ELISA kits purchased from Invitrogen according to the manufacturer’s instructions (Human Amyloid beta 40 ELISA Kit, KHB3481; Ultrasensitive Human Amyloid Beta 42 ELISA Kit, KHB3544; Amyloid beta 42 Mouse ELISA Kit, KMB3441; Amyloid beta 40 Mouse ELISA Kit, KMB3481).

### Caspase-3 activity assay

Caspase-3 activity was measured using an activity assay kit (Beyotime, C1116) according to the manufacturer’s protocol. Briefly, SH-SY5Y cells in six-well culture plates were lysed using lysis buffer for 15 min on ice. After centrifugation at 16,000 × *g* for 15 min, the supernatant was collected and incubated with Ac-DEVD-pNA (2 mM, provided in the kit) at 37 °C for two hours in 96-well plates. After incubation, absorbance at 405 nm was measured and calculated.

### Flow cytometry analysis

Apoptosis was evaluated with an Annexin V-eFluor™ 450 apoptosis detection kit (Invitrogen, 88–8006-74) according to the manufacturer’s instructions. Briefly, SH-SY5Y cells were collected, washed with ice-cold PBS (pH 7.4) and centrifuged at 1000 × *g* for 5 min. The cells were resuspended in 1 × binding buffer at 1 × 10^6^ − 5 × 10^6^ cells/ml, and 100 μl of the cell suspension was incubated with 5 μl of fluorochrome-conjugated Annexin V for 15 min at room temperature. After the cells were washed and resuspended in 200 μl of 1 × binding buffer, 5 μl of 7-AAD viability staining solution was added. The stained cells were investigated by CytoFLEX LX flow cytometry (Beckman Coulter Life Sciences, Indianapolis, IN) and analyzed using Cytexpert software (Beckman Coulter Life Sciences).

### Terminal deoxynucleotidyl transferase-mediated deoxyuridine triphosphate nick end labeling (TUNEL) assay

Apoptosis of SH-SY5Y cells was evaluated by a TUNEL assay kit (Beyotime, C1089) according to the manufacturer's protocol. Briefly, SH-SY5Y cells were fixed with 4% paraformaldehyde for 30 min at room temperature and then washed twice with PBS before being incubated in 0.3% Triton X-100 for 5 min. The cells were then incubated with the TUNEL detection mixture for 60 min at 37 °C in the dark. After being washed three times with PBS, the cells were incubated with DAPI (1:1000, Beyotime, C1002) and washed three times with PBS. The cells were then visualized using a fluorescence microscope and quantified using ImageJ software.

### Electrophysiology

Mice were anesthesized with pentobarbital sodium (i.p., 90 mg/kg) and underwent transcardial perfusion with an ice-cold dissection sucrose-rich slicing solution containing (in mM) 234 sucrose, 11 glucose, 24 NaHCO_3_, 2.5 KCl, 1.25 NaH_2_PO_4_, 0.5 CaCl_2_, and 2 MgSO_4_ bubbled with 95% O_2_/5% CO_2_. The brain was promptly removed and sliced with a vibratome. Transverse hippocampal slices (250 μm) were cut at 4 °C and subsequently recovered at 37 °C for 60 min in a chamber filled with oxygenated artificial CSF (ACSF) containing (in mM) 126 NaCl, 26 NaHCO_3_, 2.5 KCl, 1.25 NaH_2_PO_4_, 2 CaCl_2_, 2 MgCl_2_, and 10 glucose equilibrated with 95% O_2_/5% CO_2_. One hour later, slices in the holding chamber were placed at room temperature and ready for recording.

The slices were subsequently transferred to a recording chamber. A concentric circular stimulating electrode was positioned in the Schaffer collateral (SC) to deliver electrical stimuli, while a glass microelectrode filled with ACSF was placed in the stratum radiatum region of CA1 to record field excitatory postsynaptic potentials (fEPSPs). Input‒output (I/O) curves were established by recording fEPSPs and their corresponding stimulus intensities (0.05, 0.1, 0.15, 0.2, 0.25, 0.3, and 0.35 mA). fEPSPs were evoked with an intensity (1 ms) that elicited ~ 50% of the maximum amplitude at 0.05 Hz. Long-term potentiation (LTP) was induced with high-frequency stimulation comprising two consecutive trains (1 s) of stimuli at 100 Hz, separated by a 20-s interval. Potentiation was calculated as the percent increase in the average fEPSP slopes during the last 10 min normalized to the average of the baseline slopes. The data were acquired with Clampex 10.2 software (Molecular Devices, Sunnyvale, CA) and analyzed with Clampfit 10.2 (Molecular Devices).

### Statistical analysis

All statistical analyses were conducted using GraphPad Prism 7, and data are presented as means ± standard errors of the means (SEMs). Datasets that passed the Shapiro–Wilk test for normality were analyzed by using an unpaired* t* test with two-tailed analysis, or one-way or two-way analysis of variance (ANOVA) with Tukey’s multiple comparison tests for multiple comparisons. The Mann–Whitney test and Kruskal–Wallis test with Dunn's multiple comparison tests were used for nonparametric statistical analysis. *P* < 0.05 was considered as statistically significant.

## Results

### Expression of CD2AP in mice

**Fig. 1:**
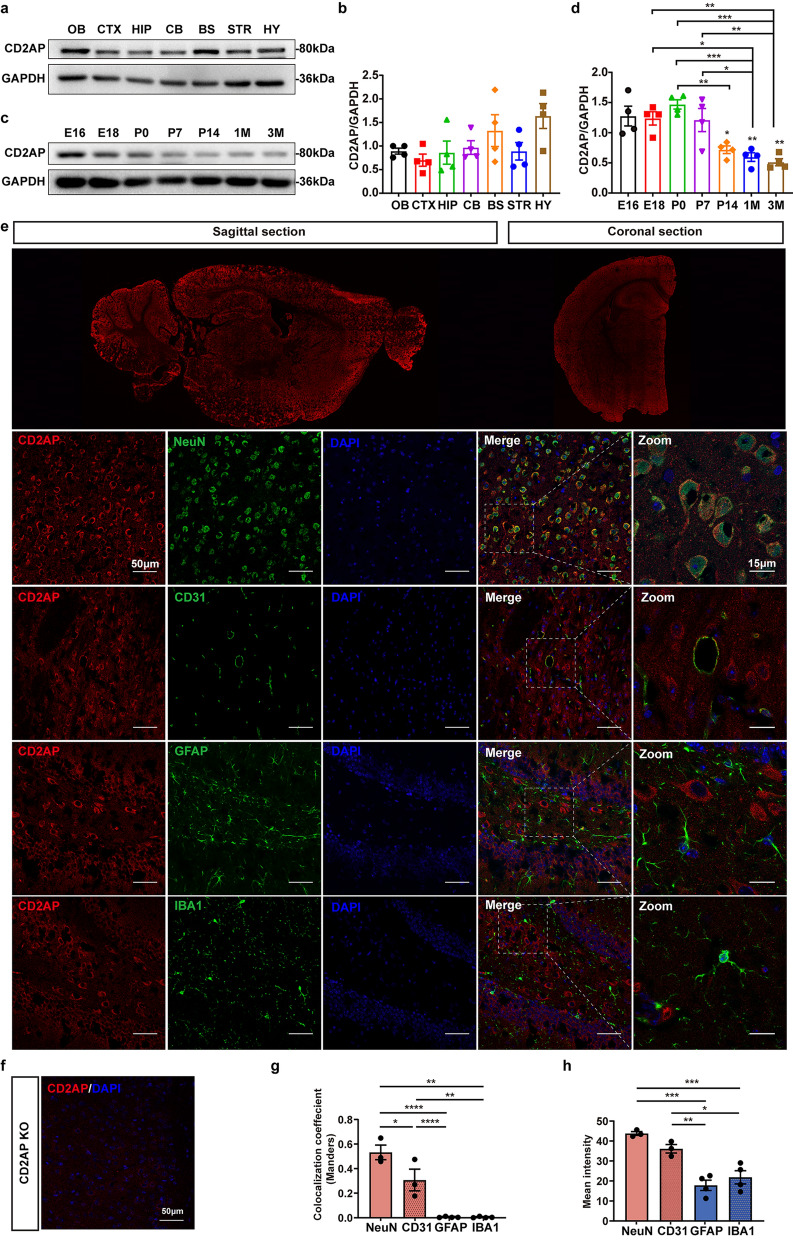
Expression of CD2AP in the brains of mice. **a**, **b** Immunoblots showed protein expression of CD2AP in the different mouse brain regions of 3-month-old mice. OB, Olfactory bulb; CTX, Cerebral cortex; HIP, hippocampus; CB, cerebellum; BS, brainstem; STR, striatum; HY, hypothalamus. **c**, **d** Immunoblots showed protein expression of CD2AP in whole-brain lysates from mice at the indicated stages of development. **e** Representative confocal images for CD2AP, NEUN, CD31, GFAP, IBA1 and DAPI in mouse cortex and hippocampus from 3-month-old mice. **f** Immunofluorescence image of CD2AP in the 1-month-old KO mouse brain as a negative control. **g** Quantitative analysis of fluorescence colocalization using Mander’s overlap coefficient methods. **h** The mean fluorescence intensity of CD2AP within the boundaries of the different cellular markers. All data are presented as mean ± SEM. One-way ANOVA with Turkey’s multiple comparison tests for multiple comparisons (**b**, **d**, **g, h**). *P < 0.05, **P < 0.01

To explore the expression pattern of CD2AP in vivo, we measured the protein levels of CD2AP in various organs from 3-month-old mice and found that CD2AP was highly expressed in the lung and testis; moderately expressed in the brain, spinal cord, heart, spleen, stomach, intestine and kidney; and expressed at low levels in the liver, muscle and eyes (Fig. S1). A survey of CD2AP expression in different brain regions by Western blotting confirmed that CD2AP is widely expressed in the brains of 3-month-old mice (Fig. 1a, b). Interestingly, the protein level of CD2AP in mouse brains gradually decreased with postnatal development (Fig. 1c, d). Immunofluorescence staining also showed that CD2AP was widely expressed in the mouse brain, and the cellular distribution of CD2AP was further assessed by confocal microscopy (Fig. 1e, f). In the cortex and hippocampus of the mouse brain, CD2AP was mainly expressed in neurons and vascular endothelial cells but barely expressed in astrocytes or microglia (Fig. 1g, h). These findings suggest a possible role of CD2AP in neuronal function.

### Generation of neuron-specific *Cd2ap* knockout mice

**Fig. 2:**
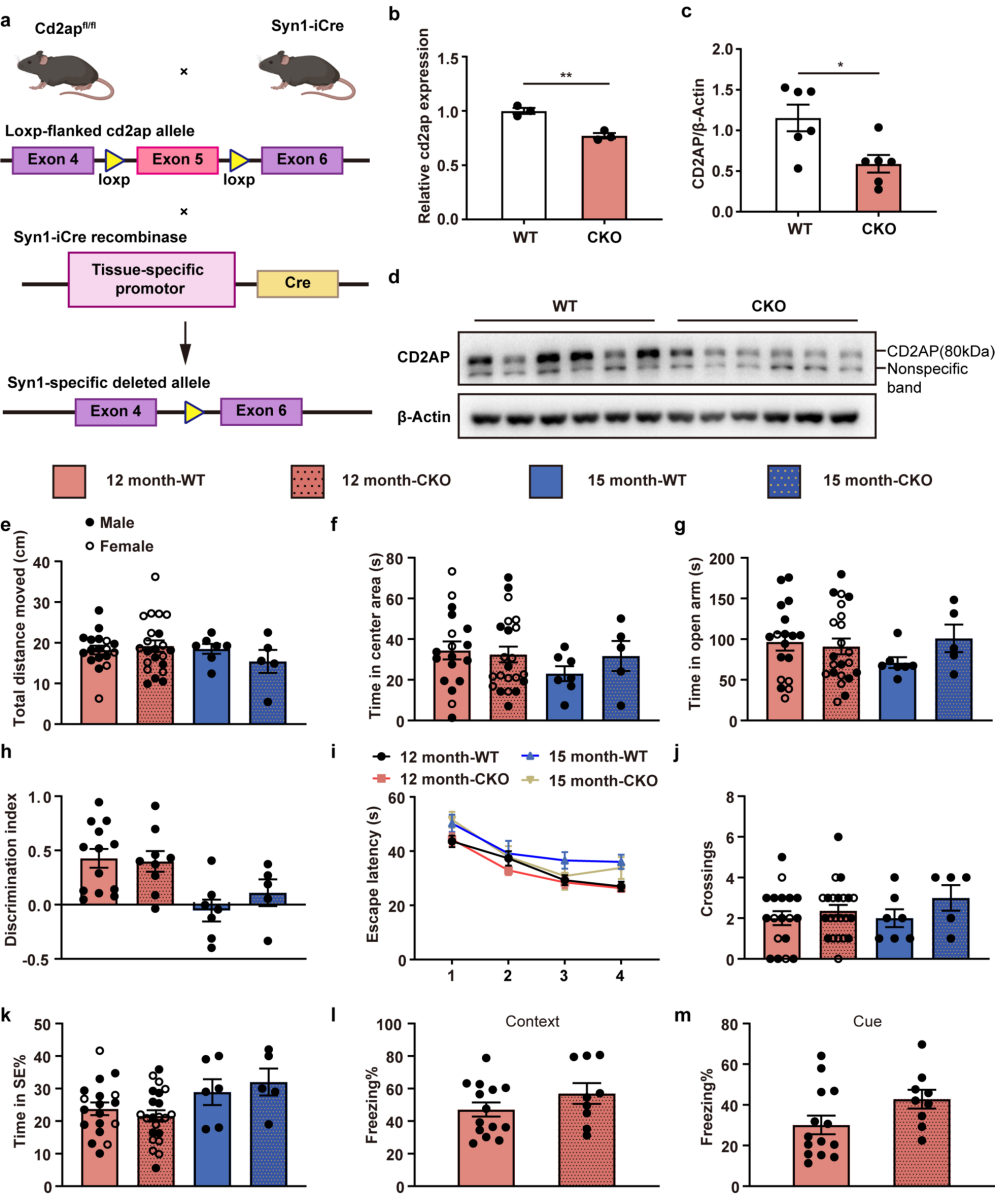
Neuronal *Cd2ap* deletion mice were not cognitively impaired. **a** Scheme of the generation of a conditional *Cd2ap* knockout mouse line. **b** RT-PCR analysis of CD2AP mRNA levels in whole-brain lysates from 12-month-old WT and CKO mice. **c**, **d** Immunoblots showing decreased protein expression of CD2AP in the brains of 12-month-old CKO mice compared to age-matched WT mice. **e**, **f** No significant difference in total distance moved or time spent in the center area was observed in the open field test. *n* = 18 (12 month-WT, female *n* = 5, male *n* = 13), *n* = 22 (12 month-CKO, female *n* = 13, male *n* = 9), *n* = 7 (15 month-WT, all male), *n* = 5 (15 month-CKO, all males). **g** No significant difference in time spent in the open arm was observed in the elevated plus maze. *n* = 18 (12 month-WT, female *n* = 5, male *n* = 13), *n* = 22 (12 month-CKO, female *n* = 13, male *n* = 9), *n* = 7 (15 month-WT, all males), *n* = 5 (15 month-CKO, all males). **h** There was no difference in preference for the novel object between WT and CKO both at 12 months and 15 months. *n* = 13 (12 month-WT, all male), *n* = 9 (12 month-CKO, all male), *n* = 7 (15 month-WT, all male), *n* = 5 (15 month-CKO, all males). **i-k** In the Morris Water maze, CKO and WT mice showed no difference in escape latency, the number of crossings of the platform or time spent in the target area. *n* = 18 (12 month-WT, female *n* = 5, male *n* = 13), *n* = 22 (12 month-CKO, female *n* = 13, male *n* = 9), *n* = 7 (15 month-WT), *n* = 5 (15 month-CKO). **l**, **m** In the fear conditioning test, 12-month-old CKO mice showed a similar freezing time to WT mice. *n* = 14 (WT, all male), *n* = 9 (CKO, all male). All data are presented as mean ± SEM. Unpaired *t*-test with two-tailed analysis was conducted separately for 12- and 15-month-old mice. *P < 0.05, **P < 0.01

Consistent with previous studies [17], global knockout of *Cd2ap* resulted in low body weight and early death at 6–7 weeks of age due to renal failure (Fig. S2a–c). For analysis of the functional role of neuronal CD2AP in AD, we generated mice with conditional neuronal *Cd2ap* knockout (hereinafter referred to as CKO mice) by crossing *Cd2ap*^fl/fl^ mice and Syn1-iCre mice (Fig. 2a). CKO mice bearing the flox and Syn1-iCre transgenes were identified via PCR analysis of mouse DNA. The CD2AP mRNA (containing exon 5) and protein levels were largely decreased in the brains of 12-month-old CKO mice (Fig. 2b–d). Compared with age- and sex-matched wild-type (WT) mice, 12-month-old CKO mice had no significant difference in body weight and developed normally (Fig. S2d).

### Neuron-specific *Cd2ap*-knockout mice do not show cognitive impairment at 15 months of age

To determine whether the deletion of *Cd2ap* in neurons directly impairs cognitive function in mice, 12- and 15-month-old CKO mice were subjected to a series of behavioral tests. Locomotor function and anxiety were evaluated in the open field test and elevated plus maze. In the CKO group, the total distance traveled and percentage of time spent in the center area in the open field test as well as the time spent in the open arm in the elevated plus maze were similar to those of WT mice at each age, indicating normal motor function and anxiety levels (Fig. 2e–g). We then performed the novel object recognition test and Morris maze test to assess episodic and spatial memory. CKO and WT mice both spent significantly more time exploring the novel object than the familiar object at 12 months of age, but both CKO and WT mice failed to show a preference for the novel object at 15 months of age (Fig. 2h). In the Morris water maze test, all mice showed reduced escape latency, indicating effective learning of the task (Fig. 2i). In the probe trial, no significant differences were observed in the number of platform crossings or the time spent in the target quadrant (Fig. 2j, k). Owing to the high stimulation, the mice were not subjected to fear conditioning tests after the Morris water maze test to avoid underlying adverse effects. Thus, only 12-month-old mice were assessed in the fear conditioning test due to the limited number of 15-month-old mice. In the fear conditioning test, there was no significant difference in context or cue freezing between the CKO and WT mice (Fig. 2l, m). Taken together, these behavioral results indicated that neuron-specific *Cd2ap* depletion does not lead to cognitive impairment. Silver staining revealed no obvious neurofibrillary tangles in the brains of 15-month-old CKO mice (Fig. S2e). ELISA results for mouse-derived Aβ showed that the levels of soluble and insoluble Aβ42, as well as the Aβ42/Aβ40 ratio, in the CKO group were not significantly different from those in the WT group (Fig. S2f).

### Neuronal *Cd2ap* knockout exacerbates cognitive function and tau pathological features in APP/PS1 mice

**Fig. 3:**
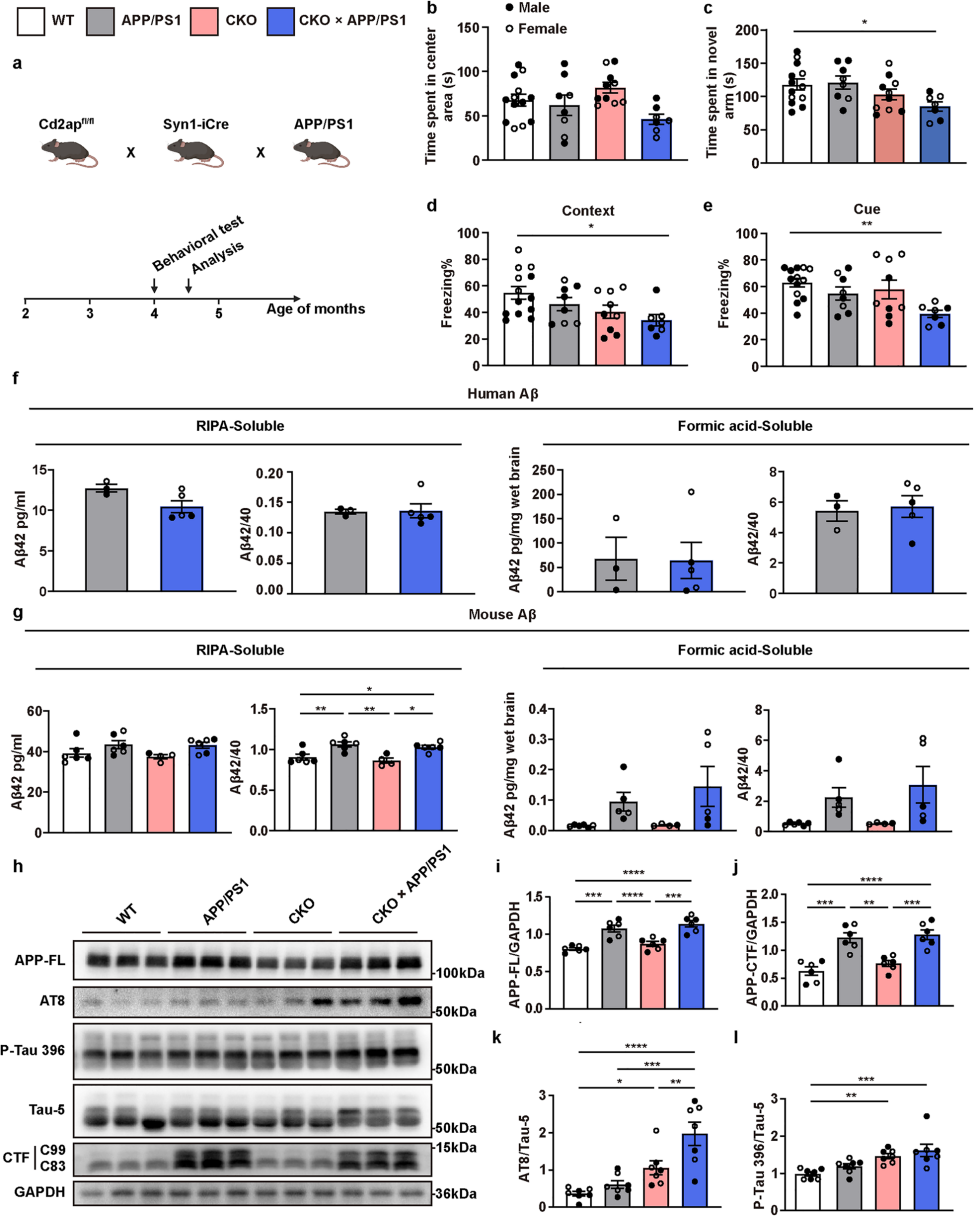
Neuronal *Cd2ap* deletion aggravated cognitive function and pathological features in APP/PS1 mice. **a** Scheme of the experimental mouse timeline. Briefly, a series of behavioral tests were performed in 4-month-old mice, and subsequent pathological analyses were conducted in 4.5-month-old mice. **b** No significant difference in time spent in the center area was observed in the open field test. *n* = 13 (WT, female *n* = 5, male *n* = 8), *n* = 8 (APP/PS1, female *n* = 3, male *n* = 5), *n* = 10 (CKO, female *n* = 5, male *n* = 5), *n* = 7 (CKO × APP/PS1, female *n* = 4, male *n* = 3). **c** CKO × APP/PS1 mice spent less time in the novel arm in the Y-maze novel arm preference test compared to WT mice. *n* = 13 (WT, female *n* = 5, male *n* = 8), *n* = 8 (APP/PS1, female *n* = 3, male *n* = 5), *n* = 10 (CKO, female *n* = 5, male *n* = 5), *n* = 7 (CKO × APP/PS1, female *n* = 4, male *n* = 3). **d**, **e** CKO × APP/PS1 mice showed significantly decreased contextual and cue-related freezing compared to WT mice. *n* = 13 (WT, female *n* = 5, male *n* = 8), *n* = 8 (APP/PS1, female *n* = 3, male *n* = 5), *n* = 9 (CKO, female *n* = 5, male *n* = 4), *n* = 7 (CKO × APP/PS1, female *n* = 4, male *n* = 3). **f** ELISA analysis of Aβ showed that neuronal *Cd2ap* deletion had no obvious influence on human Aβ level. *n* = 3 (APP/PS1, female *n* = 1, male *n* = 2), *n* = 5 (CKO × APP/PS1, female *n* = 3, male *n* = 2). **g** ELISA analysis of Aβ showed that neuronal *Cd2ap* deletion had no obvious influence on murine Aβ level. *n* = 6 (WT, female *n* = 2, male *n* = 4), *n* = 6 (APP/PS1, female *n* = 3, male *n* = 3), *n* = 4 (CKO, female *n* = 3, male *n* = 1), *n* = 6 (CKO × APP/PS1, female *n* = 4, male *n* = 2). **h-j** In 4.5-month-old mice, Immunoblots revealed that neuronal *Cd2ap* deletion had no obvious influence on the full-length APP (APP-FL) and APP-CTF proteins. *n* = 6 (WT, female *n* = 3, male *n* = 3), *n* = 6 (APP/PS1, female *n* = 3, male *n* = 3), *n* = 6 (CKO, female *n* = 3, male *n* = 3), *n* = 6 (CKO × APP/PS1, female *n* = 3, male *n* = 3). **k, l** In 4.5-month-old mice, immunoblots revealed that neuronal *Cd2ap* deletion led to significantly increased ptau202/205 (AT8) and p-tau396 level, especially in the CKO × APP/PS1 mice. *n* = 7 (WT, female *n* = 3, male *n* = 4), *n* = 7 (APP/PS1, female *n* = 3, male *n* = 4), *n* = 7 (CKO, female *n* = 4, male *n* = 3), *n* = 7 (CKO × APP/PS1, female *n* = 4, male *n* = 3). All data are presented as mean ± SEM. Unpaired *t*-test with two-tailed analysis (**f)**, one-way ANOVA with Turkey’s multiple comparison tests for multiple comparisons (**b-e, g, i, j, k)**, Kruskal–Wallis tests with Dunn’s multiple comparison tests (**l)**. *P < 0.05, **P < 0.01, ***P < 0.001, ****P < 0.0001

To further investigate the role of neuronal CD2AP in AD, we crossed APP/PS1 transgenic mice (APPSwe, PSEN1dE9) with CKO mice (Fig. 3a). Behavioral tests were performed on 4-month-old mice. The open field test showed no significant differences in simple motor activity or anxiety levels among the four groups of mice (Fig. 3b). The Y-maze novel arm preference test showed that the CKO × APP/PS1 mice spent less time in the novel arm than did the WT mice (Fig. 3c), indicating spatial and working memory impairment in the CKO × APP/PS1 mice at four months of age. We then performed a fear conditioning test to further confirm the importance of CD2AP in hippocampus-dependent learning and memory. As expected, the freezing time of the CKO × APP/PS1 mice was significantly decreased in both contextual and cued tests compared with that of the WT group, while the APP/PS1 and CKO mice did not show obvious impairment in the fear conditioning tests (Fig. 3d, e). These findings indicated that neuronal *Cd2ap* knockout leads to an earlier age of onset in APP/PS1 mice.

Subsequently, pathological tests were performed on 4.5-month-old mice. To investigate whether *Cd2ap* deletion accelerated the onset of AD by increasing Aβ accumulation, we performed ELISA to measure the levels of RIPA-soluble and RIPA-insoluble (formic-acid soluble) Aβ. Interestingly, neuronal *Cd2ap* knockout barely increased human Aβ levels and even led to a decreased tendency in RIPA-soluble Aβ42 levels in the APP/PS1 mice (Fig. 3f). Moreover, the transgenic expression of APP/PS1 led to an elevated soluble murine Aβ42/Aβ40 ratio and a tendency toward increased insoluble murine Aβ42 and Aβ42/Aβ40, while neuronal *Cd2ap* knockout barely influenced the level of murine Aβ42 (Fig. 3g). Immunofluorescence analysis revealed that neuronal CD2AP deficiency had no effect on Aβ42, pan-Aβ, or amyloid fibril levels (Fig. S3a–d). We also performed Western blot analysis to detect the full-length APP protein and its proteolytic products carboxy terminal fragment (CTFs), and found that *Cd2ap* knockout did not exert an obvious effect on the APP protein in vivo (Fig. 3h–j). Amyloid plaques were then visualized via immunofluorescence and quantified using ImageJ software. Although very small and rare because of the young age of the mice, amyloid plaques were still detected in every brain section from the APP/PS1 and CKO × APP/PS1 mice, and there was no significant increase in plaque number in the CKO × APP/PS1 mice (Fig. S3e, f).

We next determined the level of tau phosphorylation, which is one of the most important pathogeneses of AD. As reported in previous studies, hyperphosphorylated tau can be detected in 8-month-old APP/PS1 mice [21]. In the present study, hyperphosphorylated tau was not obviously increased in the 4.5-month-old APP/PS1 mice, but increased significantly in the age-matched CKO and CKO × APP/PS1 mice (Fig. 3h, k, l, Fig. S4). These findings indicated that neuronal *Cd2ap* knockout triggered tau hyperphosphorylation but exerted little effect on Aβ levels, which was consistent with our previous clinical observation that CD2AP had a stronger association with CSF tau levels than with Aβ levels [13]. Additionally, immunofluorescence analysis indicated that the numbers of Iba1-positive and GFAP-positive cells were not significantly changed in both 4.5-month-old CKO and CKO × APP/PS1 mice, indicating that neuronal *Cd2ap* deletion does not influence the activation of microglia and astrocytes (Fig. S5).

### Proteomic analysis of *Cd2ap* knockout mice

**Fig. 4:**
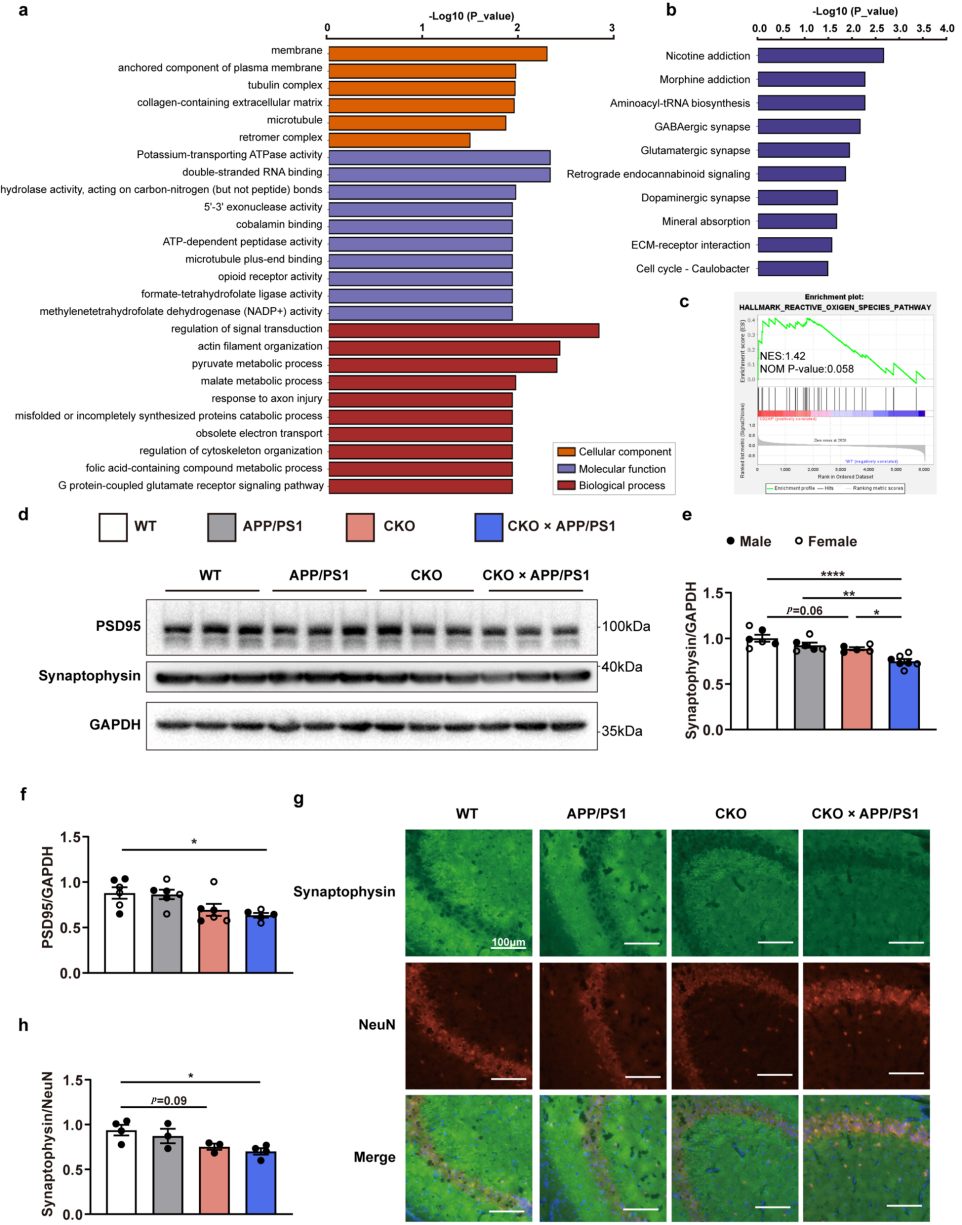
Proteomic analyses in *Cd2ap* knockout mice and neuronal *Cd2ap* deletion led to synaptic loss. **a** GO annotation analysis based on differentially expressed proteins was presented as the top six in cellular component (CC), and the top ten in biological process (BP) and molecular function (MF). **b** The top ten enriched pathways from KEGG pathway analysis of differentially expressed proteins. **c** Gene set enrichment analysis (GSEA) revealed that the reactive oxygen species pathway was upregulated in the *Cd2ap* knockout group. **d**, **e** Immunoblots showed a decreased synaptophysin level in the 4.5-month-old CKO and CKO × APP/PS1 mice. *n* = 6 (WT, female *n* = 3, male *n* = 3), *n* = 6 (APP/PS1, female *n* = 3, male *n* = 3), *n* = 5 (CKO, female *n* = 3, male *n* = 2), *n* = 7 (CKO × APP/PS1, female *n* = 4, male *n* = 3). **f** Immunoblots showed a decreased PSD95 level in the 4.5-month-old CKO × APP/PS1 mice. *n* = 6 (WT, female *n* = 3, male *n* = 3), *n* = 6 (APP/PS1, female *n* = 3, male *n* = 3), *n* = 6 (CKO, female *n* = 3, male *n* = 3), *n* = 5 (CKO × APP/PS1, female *n* = 2, male *n* = 3). **g**, **h** Immunofluorescence analysis showed significantly decreased synaptophysin in the hippocampus in 4.5-month-old CKO and CKO × APP/PS1 mice. *n* = 4 (WT, all male), *n* = 3 (APP/PS1, all male), *n* = 3 (CKO, all male), *n* = 4 (CKO × APP/PS1, all male). All data are presented as mean ± SEM. One-way ANOVA with Turkey’s multiple comparison tests for multiple comparisons (**e**, **h**), Kruskal–Wallis tests with Dunn’s multiple comparison tests (**f**). *P < 0.05, **P < 0.01, ***P < 0.001, ****P < 0.0001

Proteomic analysis based on tandem mass tags (TMTs) was performed on the brains of 1-month-old WT and *Cd2ap* knockout mice. Compared with the WT group, there were 719 differentially expressed proteins (DEPs) in the *Cd2ap* knockout group, including 267 upregulated and 452 downregulated proteins (Additional file 2: Table S2). Gene Ontology (GO) annotation analysis was performed on both upregulated and downregulated proteins, and the top six GO terms in the cellular component category and the top 10 in the biological process and molecular function categories are shown in Fig. 4a. Most DEPs originated from the membrane and are involved in the regulation of signal transduction and actin filament bundle assembly. Moreover, Kyoto Encyclopedia of Genes and Genomes (KEGG) pathway analysis was performed to further investigate the functions of the DEPs. The top 10 KEGG pathways, including GABAergic synapse, glutamatergic synapse and dopaminergic synapse, are presented in Fig. 4b, which indicated that the DEPs are highly enriched in pathways associated with synaptic function. Gene set enrichment analysis of the DEPs revealed that the reactive oxygen species pathway was promoted in the *Cd2ap* knockout group (Fig. 4c).

### Neuronal deletion of *Cd2ap* leads to synaptic loss

A reduction in synaptophysin, which is an important marker of AD progression, has been well validated in AD brains and interpreted as evidence for synaptic loss [22–24]. As TMT-based proteomic analysis indicated that *Cd2ap* knockout led to alterations in synaptic function, we next performed immunoblotting and immunofluorescence to measure the level of synaptophysin in the brains of 4.5-month-old mice. Immunoblot analysis revealed a decreasing trend for the synaptophysin level in the brains of the CKO mice, while the synaptophysin protein level was significantly decreased in the CKO × APP/PS1 mice (Fig. 4d, e). Moreover, immunoblot results showed a significant decrease of PSD95 level in the brains of 4.5-month-old CKO × APP/PS1 mice, but no significant decrease was observed in 4.5-month-old CKO mice (Fig. 4d, f). Immunofluorescence staining showed a trend toward decreased synaptophysin levels in 4.5-month-old CKO mice and a significant reduction in synaptophysin levels in CKO × APP/PS1 mice (Fig. 4g, h). These results indicate that neuronal CD2AP deficiency decreases synaptophysin protein levels in the mouse brain, and the decrease is more pronounced in the CKO × APP/PS1 mice.

**Fig. 5:**
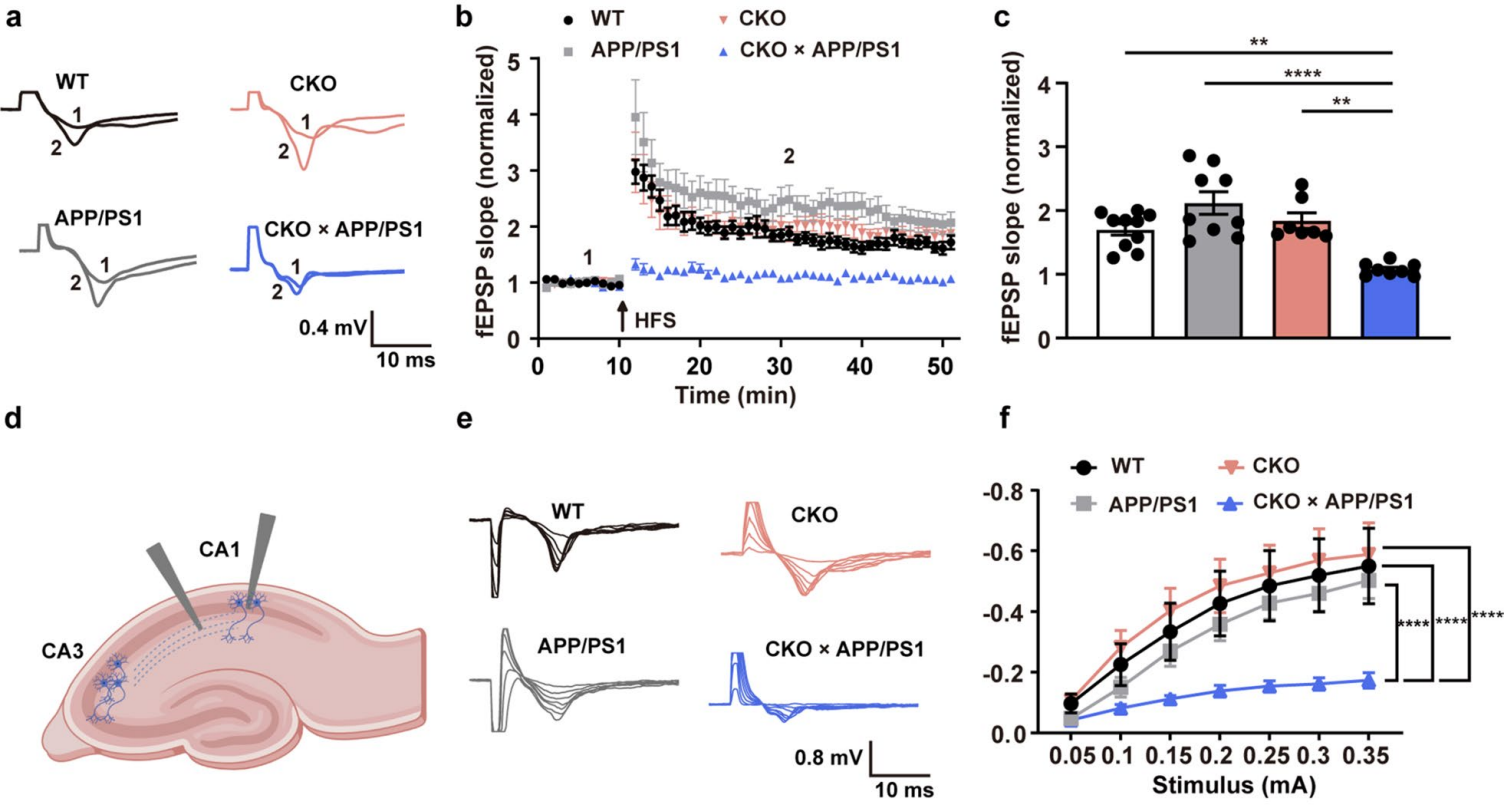
Neuronal *Cd2ap* deletion led to synaptic deficits in 4.5-month-old APP/PS1 mice. **a** Representative traces of fEPSP during LTP recording. **b** Time-course of fEPSP slopes during LTP recording. **c** The normalized average fEPSP slopes during the last 10 min. *n* = 10 from 3 mice in WT group, *n* = 9 from 3 mice in APP/PS1 group, *n* = 7 from 3 mice in CKO group, *n* = 8 from 3 mice in CKO × APP/PS1 group, one-way ANOVA with Turkey’s multiple comparison tests for multiple comparisons. **d** Schematic diagram showing the localization of stimulating and recording electrodes in the hippocampal slice. **e** Representative I/O curves for fEPSPs recorded in hippocampal CA1 subregion from 4 groups. **f** Quantitative analyses of I/O curves. *n* = 10 from 3 male mice in WT group, *n* = 9 from 3 male mice in APP/PS1 group, *n* = 9 from 3 male mice in CKO group, *n* = 9 from 3 male mice in CKO × APP/PS1 group, two-way ANOVA. HFS: High-frequency stimulation. All data are presented as mean ± SEM. *P < 0.05, **P < 0.01, ***P < 0.001, ****P < 0.0001

To further determine the effect of CD2AP on synaptic functions, we next recorded LTP and I/O curves in hippocampal slices from 4.5-month-old mice. As shown in Fig. 5, neuronal *Cd2ap* deletion alone did not alter LTP or basal synaptic transmission, as measured by I/O curves. However, neuronal *Cd2ap* deletion in APP/PS1 mice significantly reduced LTP and basal synaptic transmission. These findings indicated that neuronal *Cd2ap* deletion triggers tau pathology and that coexpression of the APP/PS1 mutant transgenes accelerates synaptic loss and deficits.

### Neuronal-specific *Cd2ap* knockout activates p38 MAPK signaling and apoptosis

**Fig. 6:**
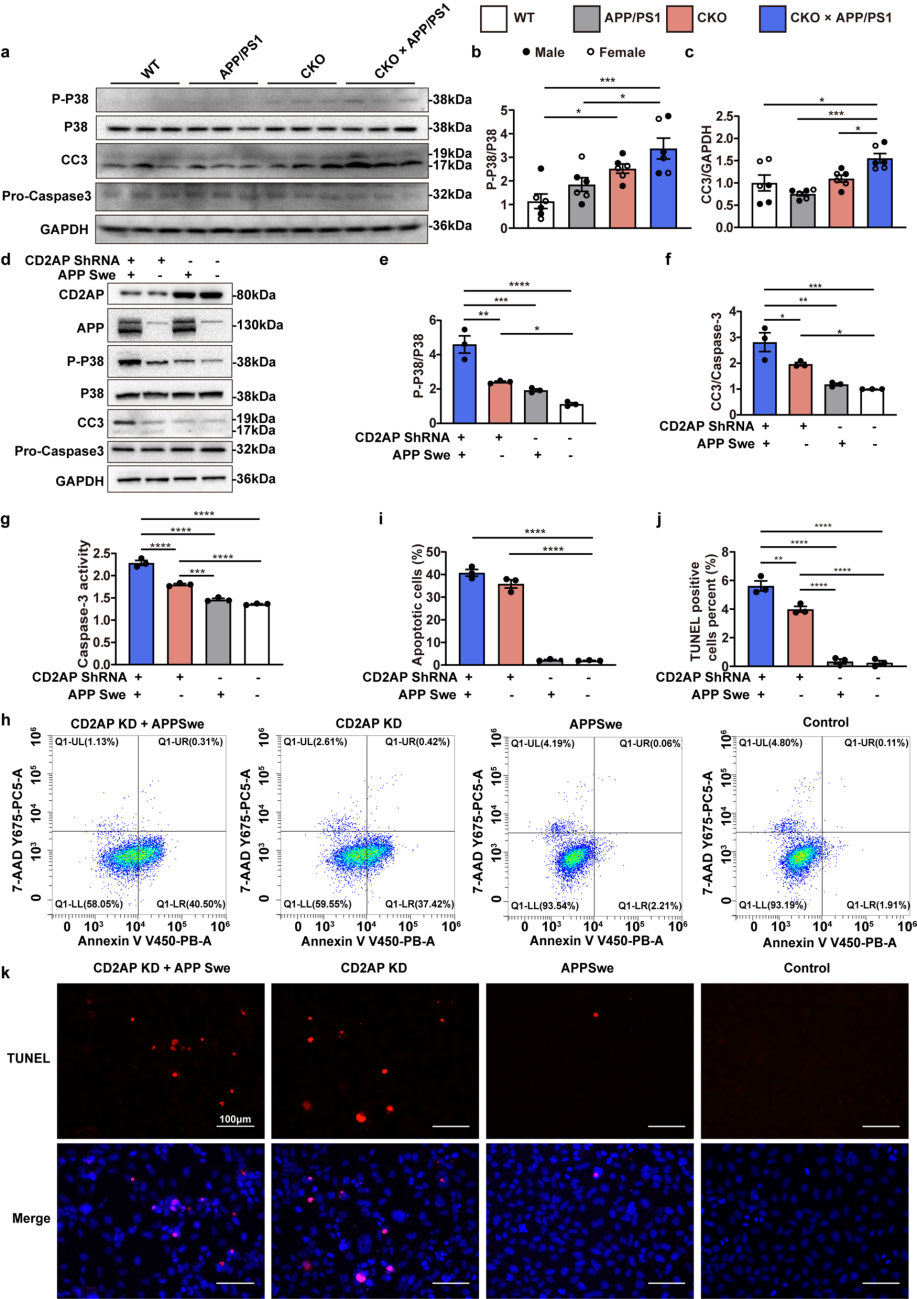
Neuronal *Cd2ap* knockout activated p38 MAPK signaling and apoptosis. **a**–**c** Immunoblots showed significantly increased phosphorylated p38 MAPK in 4.5-month-old CKO and CKO × APP/PS1 mice and significantly increased CC3 level in 4.5-month-old CKO × APP/PS1 mice. *n* = 6 (WT, female *n* = 3, male *n* = 3), *n* = 6 (APP/PS1, female *n* = 3, male *n* = 3), *n* = 6 (CKO, female *n* = 3, male *n* = 3), *n* = 6 (CKO × APP/PS1, female *n* = 3, male *n* = 3). **d**–**f** Immunoblots showed significantly increased p-p38 MAPK and CC3 levels in *CD2AP* stable knockdown cells and especially in KD cells with APPSwe co-expression. **g** Knockdown of *CD2AP* with APPSwe expression significantly increased caspase-3 activity in SH-SY5Y cells. **h**, **i** Analysis of flow cytometry showed that the number of apoptotic cells was significantly increased in *CD2AP* knockdown cells and especially in *CD2AP* knockdown cells with APPSwe co-expression. The vertical axis represented the cells labeled with 7-AAD and the horizontal axis indicated those stained with Annexin V (eFluor™ 450). The percentage of cells labeled with Annexin V but without 7-AAD was calculated and compared. **j**, **k** The apoptotic cells were detected with the TUNEL assay with nuclei stained with DAPI. The percentage of cells was quantified in different groups. All data are presented as mean ± SEM, *n* = 3 per group (**d**–**k**). One-way ANOVA with Turkey’s multiple comparison test for multiple comparisons. *P < 0.05, **P < 0.01, ***P < 0.001, ****P < 0.0001

Because hyperphosphorylated tau was observed in 4.5-month-old CKO mice, several major kinases involved in tau phosphorylation were analyzed, including p38 MAPK, CDK5 and glycogen synthase-3β (GSK-3β)[25, 26]. The phosphorylation of p38 MAPK was significantly higher in the 4.5-month-old CKO and CKO × APP/PS1 groups compared to the WT group, while no obvious increase was observed in the APP/PS1 group (Fig. 6a, b). In contrast, there were no obvious changes in the levels of CDK5, p35 (an activator of CDK5) or phospho-GSK-3β-S9 in the CKO or CKO × APP/PS1 mice (Fig. S6a − d). As previously reported, both Aβ toxicity and tau hyperphosphorylation can induce activation of the p38 MAPK stress pathway, which in turn exacerbates tau hyperphosphorylation [27]. In addition, the activation of the p38 MAPK stress pathway can induce apoptosis, which has been well established both in vitro and in vivo [26, 28]. We then assessed the level of cleaved caspase-3 (CC3), which is one of the most important executioners of apoptosis. Results revealed that the level of CC3 was significantly increased in the CKO × APP/PS1 mice (Fig. 6a, c). However, there was no significant increase in the levels of CC3 in the brains of the 4.5-month-old APP/PS1 and CKO mice (Fig. 6a, c). These findings suggest that neuronal *Cd2ap* knockout activates p38 MAPK kinase signaling, leading to the hyperphosphorylation of tau, which ultimately results in apoptosis accompanied by the presence of Aβ.

To further validate the above findings, we constructed a stable *CD2AP* knockdown (KD) SH-SY5Y cell line using lentiviral transduction (Fig. S6e, f). We then transfected the APP “Swedish” variant (APPSwe) plasmid into both Sh-Ctrl SH-SY5Y and Sh-CD2AP SH-SY5Y cells. Compared with the normal SH-SY5Y cells, the Sh-CD2AP cells with APPSwe overexpression showed strongly increased p38 MAPK signaling and CC3 level, while *CD2AP* knockdown without APPSwe overexpression also resulted in activation of p38 MAPK signaling and increased CC3 level (Fig. 6d–f). In addition, caspase-3 activity was increased in the Sh-CD2AP cells with and without APPSwe overexpression (Fig. 6g). However, *CD2AP* knockdown in combination with APPSwe overexpression significantly increased p38 MAPK activation and CC3 levels compared with *CD2AP* knockdown or APPSwe overexpression alone (Fig. 6d–g). To further measure the level of apoptosis, we performed Annexin V and 7-AAD double staining for flow cytometry and TUNEL staining. Flow cytometry revealed that the percentage of apoptotic cells stained with single Annexin V (indicative of early apoptosis) was significantly greater, up to 40.77% and 35.89%, in the CD2AP KD + APPSwe group and CD2AP KD group, respectively, compared to the APPSwe and NC groups (2.25% and 1.98%, respectively) (Fig. 6h, i). Moreover, TUNEL staining revealed significant increases in the percentage of apoptotic cells in the CD2AP KD + APPSwe group and the CD2AP KD group (Fig. 6j, k). These results indicated that *CD2AP* knockdown significantly activates apoptosis by increasing p38 MAPK kinase signaling and CC3 levels, especially in the presence of pathological Aβ proteins.

### Inhibition of p38 MAPK rescues learning deficits and synaptic loss in CKO × APP/PS1 mice

**Fig. 7:**
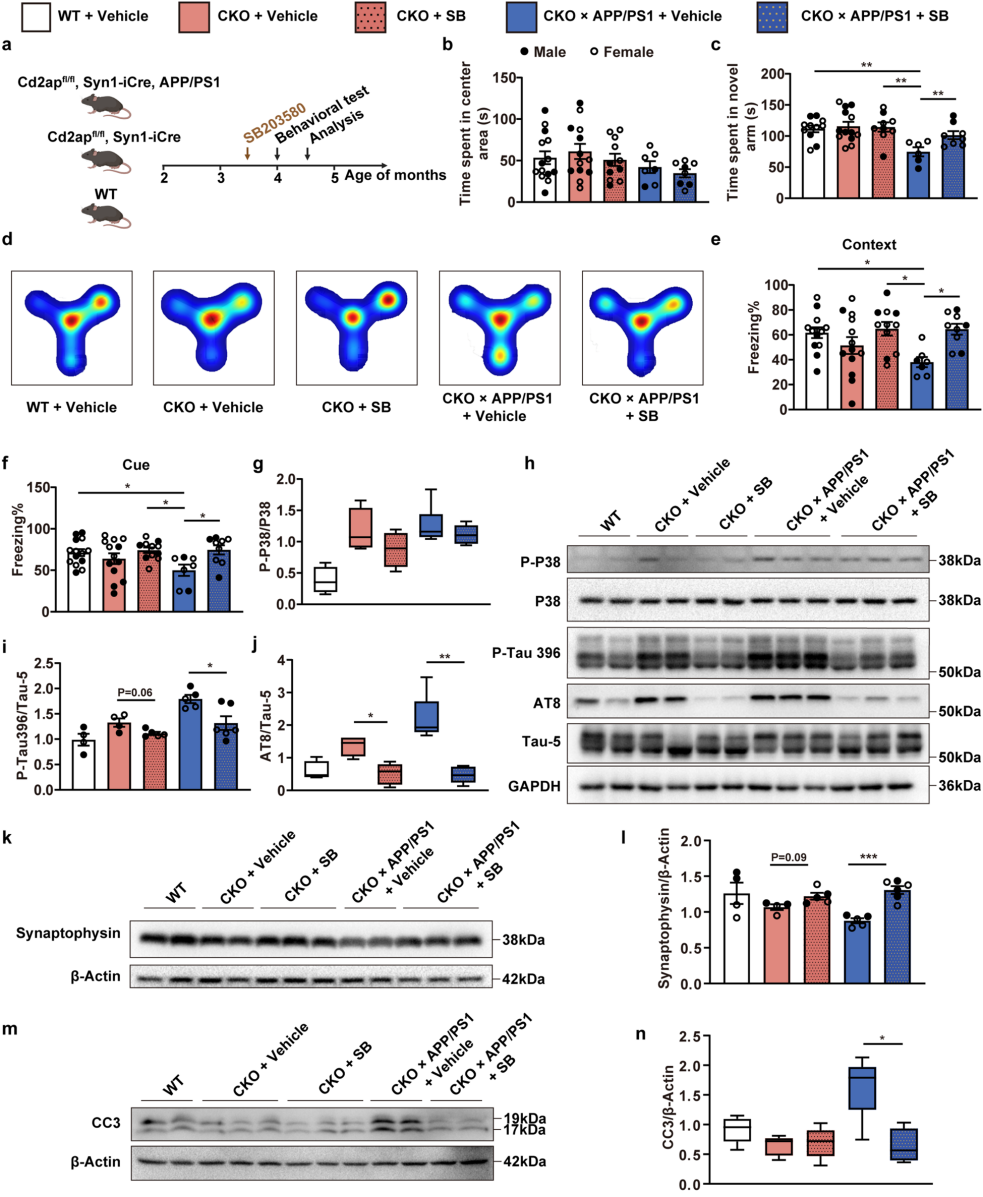
Inhibition of p38 MAPK rescued learning deficit, synaptic loss, and apoptosis in CKO × APP/PS1 mice. **a** Scheme of drug treatment and test schedules. **b** No significant difference in time spent in the center area was observed among 4-month-old mice in the open field test. *n* = 14 (WT, female *n* = 8, male *n* = 6), *n* = 13 (CKO + Vehicle, female *n* = 6, male *n* = 7), *n* = 11 (CKO + SB, female *n* = 7, male *n* = 4), *n* = 7 (CKO × APP/PS1 + Vehicle, female *n* = 5, male *n* = 2), *n* = 8 (CKO × APP/PS1 + SB, female *n* = 5, male *n* = 3). **c**, **d** In the Y-maze novel arm preference test, 4-month-old CKO × APP/PS1 mice given SB203580 treatment spent more time in the novel arm than those given the vehicle. *n* = 11 (WT, female *n* = 7, male *n* = 4), *n* = 13 (CKO + Vehicle, female *n* = 6, male *n* = 7), *n* = 9 (CKO + SB, female *n* = 6, male *n* = 3), *n* = 6 (CKO × APP/PS1 + Vehicle, female *n* = 4, male *n* = 2), *n* = 8 (CKO × APP/PS1 + SB, female *n* = 6, male *n* = 2). **e**, **f** Four-month-old CKO × APP/PS1 mice given SB203580 treatment showed significantly increased contextual and cue-related freezing time than those given the vehicle. *n* = 14 (WT, female *n* = 8, male *n* = 6), *n* = 13 (CKO + Vehicle, female *n* = 6, male *n* = 7), *n* = 11 (CKO + SB, female *n* = 7, male *n* = 4), *n* = 7 (CKO × APP/PS1 + Vehicle, female *n* = 5, male *n* = 2), *n* = 9 (CKO × APP/PS1 + SB, female *n* = 6, male *n* = 3). **g** SB203580 treatment did not obviously suppress the phosphorylated p38 MAPK in 4.5-month-old CKO and CKO × APP/PS1 mice. *n* = 4 (WT, female *n* = 2, male *n* = 2), *n* = 4 (CKO + Vehicle, female *n* = 2, male *n* = 2), *n* = 4 (CKO + SB, female *n* = 2, male *n* = 2), *n* = 6 (CKO × APP/PS1 + Vehicle, female *n* = 4, male *n* = 2), *n* = 6 (CKO × APP/PS1 + SB, female *n* = 3, male *n* = 3) **h**–**j** SB203580 treatment significantly decreased the hyperphosphorylation of tau in 4.5-month-old CKO and CKO × APP/PS1 mice, *n* = 4 (WT, female *n* = 2, male *n* = 2), *n* = 4 (CKO + Vehicle, female *n* = 2, male *n* = 2), *n* = 5 (CKO + SB, female *n* = 2, male *n* = 3), *n* = 5 (CKO × APP/PS1 + Vehicle, female *n* = 3, male *n* = 2), *n* = 6 (CKO × APP/PS1 + SB, female *n* = 3, male *n* = 3). **k**, **l** SB203580 treatment increased synaptophysin protein level in 4.5-month-old CKO and CKO × APP/PS1 mice. *n* = 4 (WT, female *n* = 2, male *n* = 2), *n* = 4 (CKO + Vehicle, female *n* = 2, male *n* = 2), *n* = 5 (CKO + SB, female *n* = 2, male *n* = 3), *n* = 5 (CKO × APP/PS1 + Vehicle, female *n* = 3, male *n* = 2), *n* = 6 (CKO × APP/PS1 + SB, female *n* = 3, male *n* = 3). **m**, **n** SB203580 treatment significantly decreased CC3 level in 4.5-month-old CKO × APP/PS1 mice. *n* = 5 (WT, female *n* = 3, male *n* = 2), *n* = 5 (CKO + Vehicle, female *n* = 3, male *n* = 2), *n* = 5 (CKO + SB, female *n* = 2, male *n* = 3), *n* = 5 (CKO × APP/PS1 + Vehicle, female *n* = 3, male *n* = 2), *n* = 4 (CKO × APP/PS1 + SB, female *n* = 2, male *n* = 2). Data are presented as mean ± SEM (**b**, **c**, **e f**, **i**, **l**) and box plots with median (**g**, **j**, **n**). One-way ANOVA with Turkey’s multiple comparison tests for multiple comparisons (**b**-**f**), One-way ANOVA or Kruskal–Wallis tests for detecting overall differences among multiple groups (**g**-**n**), followed by specific comparisons, including CKO + Vehicle vs. CKO + SB, and CKO × APP/PS1 + Vehicle vs. CKO × APP/PS1 + SB, using unpaired *t*-test with two-tailed analysis (**i**, **l**) and Mann–Whitney test (**g**, **j**, **n**), with Bonferroni correction applied. *P < 0.05, **P < 0.01, ***P < 0.001

To determine whether the p38 MAPK pathway is involved in the learning deficits and synaptic loss mediated by neuronal *Cd2ap* knockout in APP/PS1 mice, we treated CKO and CKO × APP/PS1 mice with the p38 MAPK inhibitor SB203580 (0.5 mg/kg, intraperitoneal injection, daily for two weeks) beginning at 3.5 months of age (Fig. 7a). After treatment, mice underwent open field tests, Y-maze novel arm preference tests and fear conditioning tests at 4 months of age. Results showed that SB203580 treatment did not affect spontaneous exploratory activity but significantly improved spatial and working memory in the CKO × APP/PS1 mice (Fig. 7b–d). In addition, the CKO × APP/PS1 mice treated with SB203580 showed a notable increase in freezing time in both contextual and cued fear tests, which further demonstrated that SB203580 reversed the learning deficits and memory impairment of CKO × APP/PS1 mice (Fig. 7e, f).

We then examined the phosphorylation levels of p38 MAPK and tau in 4.5-month-old mice of different groups. The results demonstrated that the phosphorylation level of p38 MAPK was not significantly decreased after SB203580 treatment (Fig. 7g, h), while the phosphorylated tau level was significantly decreased in both CKO and CKO × APP/PS1 mice after SB203580 treatment (Fig. 7h–j). As reported in a previous study, SB203580 inhibits the activity rather than the activation of p38 MAPK, which is consistent with our results [29]. Additionally, the level of synaptophysin was significantly increased in the treatment groups than in the vehicle groups (Fig. 7k, l). Moreover, the CC3 level in the CKO × APP/PS1 mice was significantly decreased by SB203580 treatment (Fig. 7m, n). These results indicated that SB203580 treatment significantly suppressed the hyperphosphorylation of tau, synaptic loss, and apoptosis, which further reduced the learning deficits and memory impairment in the CKO × APP/PS1 mice.

## Discussion

The association between CD2AP and an increased risk of AD has been consistently demonstrated by our team and other researchers. Despite these findings, the mechanisms underlying the CD2AP involvement in AD pathogenesis remain unclear. Evidence from in vitro studies suggests that CD2AP deficiency is a potential contributor to Aβ and tau pathology, yet it is difficult to determine its function in AD due to the lack of viable *Cd2ap* knockout mouse models that can reach adulthood. In this study, we provided the first description of the temporal and spatial expression patterns of CD2AP in mice. Our results indicated that the CD2AP protein was mainly distributed in neurons and vascular endothelial cells, and its levels decreased with age. On this basis, we generated and characterized neuron-specific *Cd2ap*-deletion mice, which were subsequently crossed with APP/PS1 mice. We found that 4-month-old CKO × APP/PS1 mice exhibited substantial cognitive deficits and memory impairment, while age-matched APP/PS1 mice were not cognitively impaired. To our knowledge, this is the first report that CD2AP deficiency has a substantial impact on the phenotypes of AD in vivo. Furthermore, we provided the first in vivo evidence that the absence of CD2AP in neurons resulted in a notable increase in tau phosphorylation, which was attributed to the activation of p38 MAPK. In addition, our results showed that CD2AP deficiency resulted in substantial synaptic loss and synaptic dysfunction, particularly in the presence of Aβ. Finally, both in vivo and in vitro analyses showed that downregulation of CD2AP increased p38 MAPK activation and CC3-mediated apoptosis, and this effect was reversed by the p38 MAPK inhibitor SB203580.

In this study, we found that CD2AP is expressed mainly in neurons and vascular endothelial cells within the adult mouse brain. Furthermore, CD2AP levels were abundant in the developing nervous system but significantly decreased following brain maturation. These results suggest that CD2AP may play an important role in brain development and degeneration. Besides, previous research has demonstrated high expression of CD2AP in dendritic cells, which are essential components of the innate immunity. CD2AP has been proposed to affect neuroinflammation by regulating neuroglial activity [30–33]. We found that the absence of CD2AP in neurons does not directly affect neuroinflammation.

Previous research has also established a link between CD2AP deficiency and tau pathology. Jansen et al. assessed the effect of various AD-associated loci, and found the *CD2AP* rs7767350 variant is associated with both decreased Aβ42 and increased p-tau in CSF [34]. Tan et al. analyzed the possible functional effects of variants in AD risk genes using clinical data and found that *CD2AP* is involved in both tauopathy and neurodegeneration rather than in amyloidosis [35]. Furthermore, immunofluorescence analysis of AD brains revealed the colocalization of CD2AP with p-tau [36]. Our previous study revealed that the *CD2AP* risk allele (C allele of rs9296559) is correlated with increased CSF t-tau and p-tau levels in patients with mild cognitive impairment [13]. Notably, the activation of p38 MAPK, which is involved in tau phosphorylation at pathogenic residues and is positively correlated with the amount of aggregated tau, has been well established in the brains of AD mouse models and patients during early stage of disease [37–40]. In this study, we reported that neuronal *Cd2ap* deletion resulted in the activation of p38 MAPK and increased tau phosphorylation, which also corroborated the role of CD2AP in tau pathology that has been reported in the *Drosophila* model of AD [14].

P38 MAPK regulates cellular responses to various extracellular stimuli[41]. In AD, p38 MAPK activation can be induced by both Aβ and oxidative stress, which further contributes to neuronal apoptosis [28, 38, 42]. A previous study reported that 9-month-old APP/PS1 mice exhibit increased levels of phosphorylated p38 (p-p38) in the hippocampus but not in the cortex [42]. The discrepancy between our results and the literature may arise from the relatively young age of the mice we used. The level of Aβ may not be sufficient to induce an imbalance in the phosphorylation of p38 MAPK. Additionally, our protein samples were derived from a mixture of cortical and hippocampal tissues, and the presence of cortical proteins may weaken the observation of p-p38 increase. Some chemotherapeutic agents induce apoptosis by activating p38 MAPK to treat cancers, and the neuroprotective effect of p38 MAPK inhibition against neuronal apoptosis has been demonstrated [40, 43]. In this study, knockdown of *CD2AP* in SH-SY5Y cells largely increased the levels of p-p38 and CC3, while the coexpression of APPSwe further increased the CC3 protein level and exacerbated apoptosis. In vivo, neuron-specific deletion of *Cd2ap* resulted in the activation of p38 MAPK, which was consistent with our in vitro observations. However, obvious neuronal apoptosis was observed only in CKO × APP/PS1 mice, rather than in APP/PS1 or CKO mice, at age 4 months, suggesting the complex mechanisms of apoptotic regulation in vivo. In addition to p38 MAPK, apoptosis in AD is regulated by various pathways, including BCL-2 family proteins, JNK and the PI3K/AKT/mTOR pathway [44, 45]. CD2AP deficiency combined with pathological Aβ leads to an imbalance in apoptotic pathways, which ultimately results in neuronal apoptosis. The important role of p38 MAPK in neuronal apoptosis in CKO × APP/PS1 mice was further validated through the antiapoptotic effect of a p38 MAPK inhibitor. These results indicate that CD2AP deficiency not only induces the activation of p38 MAPK but also increases the vulnerability of neurons to apoptotic stimuli.

It is also worthwhile to delve into the mechanisms that underlie how CD2AP deficiency leads to synaptic injury. CD2AP, a positive regulator of axonal sprouting and structural plasticity in neurons and PC12 cells [46], increases early during development, suggesting that CD2AP plays an active role in synaptic formation. CD2AP deficiency may weaken the regulation of axonal sprouting and structural plasticity, potentially affecting the structure and function of synapses. Additionally, we demonstrated that CD2AP deficiency resulted in the activation of p38 MAPK, which was previously reported to impair synaptic function [18, 47, 48]. Moreover, synaptic dysfunction in AD depends on the actions of both Aβ and tau [49–51]. On the one hand, Aβ affects synaptic trafficking and impairs LTP in a tau-dependent manner, as tau knockout protects APP/PS1 mice from memory impairment and synaptic dysfunction [52]. On the other hand, Aβ induces mitochondrial dysfunction and tau hyperphosphorylation, resulting in tau misplacement in the dendritic compartment and diminished synaptic activity [53]. Marcatti et al. showed that tau oligomers are capable of binding to human synapses and suppressing chemical LTP, which can be exacerbated by Aβ oligomers [54]. Our results revealed that both 4.5-month-old APP/PS1 and CKO mice exhibited a decreasing tendency of synaptophysin, while the level of synaptophysin was markedly decreased in the CKO × APP/PS1 mice. Moreover, CD2AP deficiency resulted in decreased synaptic plasticity in the APP/PS1 mice, which was consistent with the changes in the levels of synaptophysin and neuronal apoptosis. Both synaptophysin and synaptogyrin knockout mice are reported to exhibit no change in LTP, whereas double knockout of synaptophysin and synaptogyrin resulted in a significant decrease in synapse plasticity, suggesting partial functional redundancy of synapse proteins [55, 56]. Thus, despite the decreased synaptophysin level observed in CKO mice, they still exhibited intact LTP and I/O curves. This finding may partially explain why the CKO × APP/PS1 mice show obvious cognitive impairment as early as 4 months of age, while the CKO mice are not cognitively impaired even at 12 or 15 months of age. However, a limitation of our study is the absence of an evaluation of older CKO mice, necessitating further investigation in the future.

As described above, the role of CD2AP in amyloidogenesis is diverse and complex. Studies conducted in vitro and in vivo have yielded conflicting results regarding the effect of CD2AP deletion on Aβ aggregation. Specifically, in vitro suppression of CD2AP expression has been demonstrated to affect Aβ levels and the Aβ42/Aβ40 ratio; however, no significant impact of CD2AP on Aβ metabolism was observed in vivo [10, 12]. In this study, no significant change in Aβ levels was observed in the CKO × APP/PS1 mice, which proves that CD2AP deletion has minimal influence on the Aβ burden in vivo.

As previous studies reported, APP/PS1 mice exhibit cognitive deficits from 6 to 8 months of age and present decreased synaptophysin levels at 7–8 months [57–60]. Amyloid plaques begin to form between 4 and 6 months of age and increase in both size and number with age, and hyperphosphorylated tau can be detected at 8 months of age [21, 61]. In our study, the 4-month-old APP/PS1 mice did not exhibit notable cognitive deficits, while the 4.5-month-old APP/PS1 mice did not show obviously decreased synaptophysin or increased phosphorylated tau levels but displayed deposition of amyloid plaques, which is consistent with previous studies. Our study demonstrated that CD2AP deficiency expedites the onset of AD in APP/PS1 mice, and studies in older CD2AP × APP/PS1 mice are needed to further investigate the role of CD2AP in AD progression.

## Conclusion

Taken together, our study provides initial evidence that CD2AP deficiency exacerbates AD phenotypes and pathology through activation of the p38 MAPK pathway. These findings highlight the crucial role of CD2AP/p38 MAPK signaling in AD pathology, thus reinforcing the potential of targeting this pathway as a promising therapeutic avenue for the treatment of AD.

## Supplementary Information

**Supplementary file** **1**. **Figure S1**. Expression of CD2AP in different adult mouse tissues. **Figure S2**. The body weights of Cd2ap KO and CKO mice and pathological staining in CKO mice. **Figure S3**. No obvious difference in Aβ burden between APP/PS1 and CKO × APP/PS1 mice was observed. **Figure S4**. Neuronal Cd2ap deletion increase p-tau level in mice. **Figure S5**. Neuronal Cd2ap deletion did not influence the activation of microglia and astrocytes. **Figure S6**. Neuronal Cd2ap deletion exerted no influence in GSK-3β, CDK5, or p35 pathway. **Table S1**. Primer sequences for genotyping and qPCR.

**Supplementary file** **2**. **Table S2**. TMT-based proteomic analysis in the brains of 1-month-old WT and Cd2ap knockout mic

**Supplementary file** **3**. Uncropped full-length pictures of Western blotting membranes presented in the figures.

## Data Availability

Data are available from the corresponding authors upon reasonable request.

## Abbreviations

CD2AP: CD2-associated protein
AD: Alzheimer’s disease
MAPK: Mitogen-activated protein kinase
Aβ: β-Amyloid
TUNEL: Terminal deoxynucleotidyl transferase-mediated deoxyuridine triphosphate nick end labeling
LTP: Long-term potentiation
ANOVA: Analysis of variance
TMTs: Tandem mass tags
DEPs: Differentially expressed proteins
GO: Gene ontology
CC: Cellular component
BP: Biological process
KEGG: Kyoto encyclopedia of genes and genomes
CDK5: Cyclin kinase-5
GSK-3β: Glycogen synthase-3β
CC3: Cleaved caspase-3
