# Correction: CD2AP deficiency aggravates Alzheimer’s disease phenotypes and pathology through p38 MAPK activation

**DOI:** 10.1186/s40035-024-00464-3

**Published:** 2025-01-17

**Authors:** Yan-Yan Xue, Zhe-Sheng Zhang, Rong-Rong Lin, Hui-Fen Huang, Ke-Qing Zhu, Dian-Fu Chen, Zhi-Ying Wu, Qing-Qing Tao

**Affiliations:** 1 Department of Neurology, The Second Afliated Hospital, Zhejiang University School of Medicine and Liangzhu Laboratory, 88 Jiefang Road, Hangzhou, 310009 China; 2 National Health and Disease Human Brain Tissue Resource Center and Department of Pathology, School of Medicine, Zhejiang University, Hangzhou, 310058 China; 3 MOE Frontier Science Center for Brain Science and Brain-Machine Integration, School of Brain Science and Brain Medicine, Zhejiang University, Hangzhou, 310058 China; 4 CAS Center for Excellence in Brain Science and Intelligence Technology, Shanghai, 200031 China

**Correction: Translational Neurodegeneration (2024) 13:64** 10.1186/s40035-024-00454-5

Following publication of the original article [1], the authors reported an error in the Fig. 3h, which presented incorrect lane image of p-tau 396. The Fig. 3 is corrected from:

**Fig. 3:**
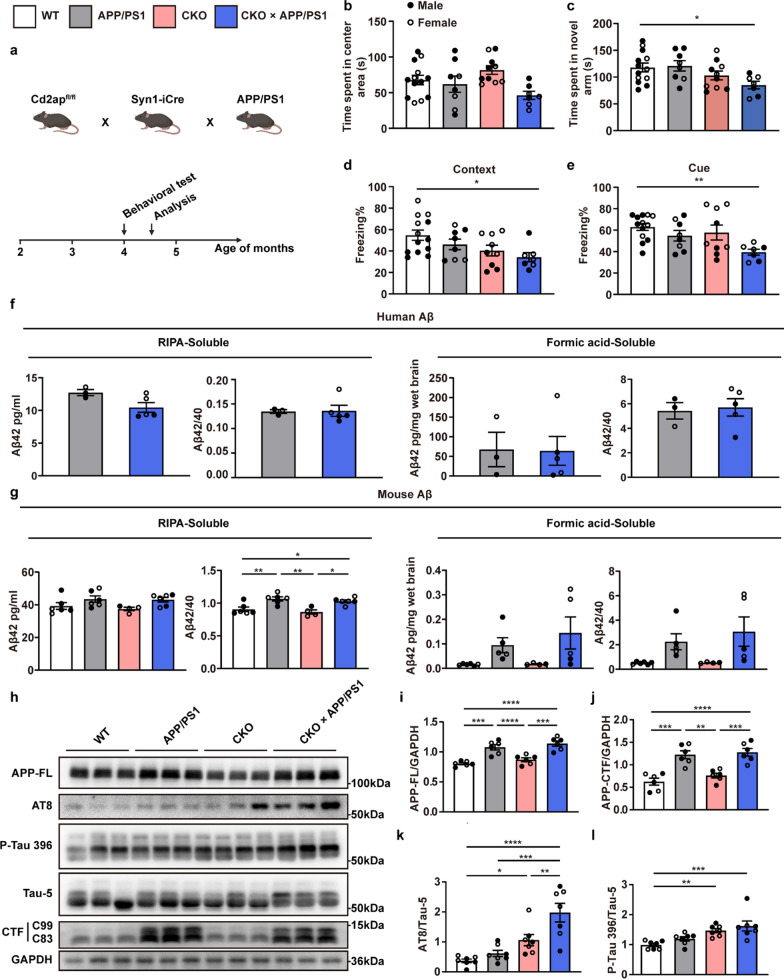
Neuronal *Cd2ap* deletion aggravated cognitive function and pathological features in APP/PS1 mice. **a** Scheme of the experimental mouse timeline. Briefly, a series of behavioral tests were performed in 4-month-old mice, and subsequent pathological analyses were conducted in 4.5-month-old mice. **b** No significant difference in time spent in the center area was observed in the open field test. *n* = 13 (WT, female *n* = 5, male *n* = 8), *n* = 8 (APP/PS1, female *n* = 3, male *n* = 5), *n* = 10 (CKO, female *n* = 5, male *n* = 5), *n* = 7 (CKO × APP/PS1, female *n* = 4, male *n* = 3). **c** CKO × APP/PS1 mice spent less time in the novel arm in the Y-maze novel arm preference test compared to WT mice. *n* = 13 (WT, female *n* = 5, male *n* = 8), *n* = 8 (APP/PS1, female *n* = 3, male *n* = 5), *n* = 10 (CKO, female *n* = 5, male *n* = 5), *n* = 7 (CKO × APP/PS1, female *n* = 4, male *n* = 3). **d, e** CKO × APP/PS1 mice showed significantly decreased contextual and cue-related freezing compared to WT mice. *n* = 13 (WT, female *n* = 5, male *n* = 8), *n* = 8 (APP/PS1, female *n* = 3, male *n* = 5), *n* = 9 (CKO, female *n* = 5, male *n* = 4), *n* = 7 (CKO × APP/PS1, female *n* = 4, male *n* = 3). **f** ELISA analysis of Aβ showed that neuronal *Cd2ap* deletion had no obvious influence on human Aβ level. *n* = 3 (APP/PS1, female *n* = 1, male *n* = 2), *n* = 5 (CKO × APP/PS1, female *n* = 3, male *n* = 2). **g** ELISA analysis of Aβ showed that neuronal *Cd2ap* deletion had no obvious influence on murine Aβ level. *n* = 6 (WT, female *n* = 2, male *n* = 4), *n* = 6 (APP/PS1, female *n* = 3, male *n* = 3), *n* = 4 (CKO, female *n* = 3, male *n* = 1), *n* = 6 (CKO × APP/PS1, female *n* = 4, male *n* = 2). **h-j** In 4.5-month-old mice, Immunoblots revealed that neuronal *Cd2ap* deletion had no obvious influence on the full-length APP (APP-FL) and APP-CTF proteins. *n* = 6 (WT, female *n* = 3, male *n* = 3), *n* = 6 (APP/PS1, female *n* = 3, male *n* = 3), *n* = 6 (CKO, female *n* = 3, male *n* = 3), *n* = 6 (CKO × APP/PS1, female *n* = 3, male *n* = 3). **k, l** In 4.5-month-old mice, Immunoblots revealed that neuronal *Cd2ap* deletion led to significantly increased ptau202/205 (AT8) and p-tau396 level, especially in the CKO × APP/PS1 mice. *n* = 7 (WT, female *n* = 3, male *n* = 4), *n* = 7 (APP/PS1, female *n* = 3, male *n* = 4), *n* = 7 (CKO, female *n* = 4, male *n* = 3), *n* = 7 (CKO × APP/PS1, female *n* = 4, male *n* = 3). All data are presented as mean ± SEM. Unpaired* t*-test with two-tailed analysis (**f)**, one-way ANOVA with Turkey’s multiple comparison tests for multiple comparisons (**b-e, g, i, j, k)**, Kruskal–Wallis tests with Dunn’s multiple comparison tests (**l)**. *P < 0.05, **P < 0.01, ***P < 0.001, ****P < 0.0001

To:

**Fig. 3:**
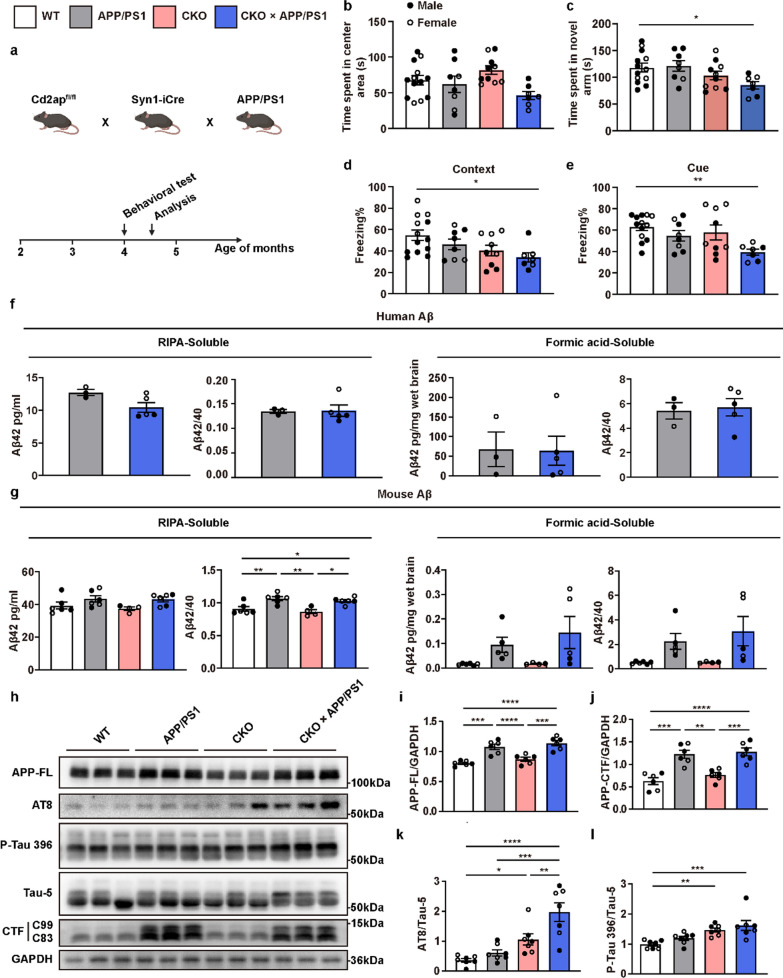
Neuronal *Cd2ap* deletion aggravated cognitive function and pathological features in APP/PS1 mice. **a** Scheme of the experimental mouse timeline. Briefly, a series of behavioral tests were performed in 4-month-old mice, and subsequent pathological analyses were conducted in 4.5-month-old mice. **b** No significant difference in time spent in the center area was observed in the open field test. *n* = 13 (WT, female *n* = 5, male *n* = 8), *n* = 8 (APP/PS1, female *n* = 3, male *n* = 5), *n* = 10 (CKO, female *n* = 5, male *n* = 5), *n* = 7 (CKO × APP/PS1, female *n* = 4, male *n* = 3). **c** CKO × APP/PS1 mice spent less time in the novel arm in the Y-maze novel arm preference test compared to WT mice. *n* = 13 (WT, female *n* = 5, male *n* = 8), *n* = 8 (APP/PS1, female *n* = 3, male *n* = 5), *n* = 10 (CKO, female *n* = 5, male *n* = 5), *n* = 7 (CKO × APP/PS1, female *n* = 4, male *n* = 3). **d, e** CKO × APP/PS1 mice showed significantly decreased contextual and cue-related freezing compared to WT mice. *n* = 13 (WT, female *n* = 5, male *n* = 8), *n* = 8 (APP/PS1, female *n* = 3, male *n* = 5), *n* = 9 (CKO, female *n* = 5, male *n* = 4), *n* = 7 (CKO × APP/PS1, female *n* = 4, male *n* = 3). **f** ELISA analysis of Aβ showed that neuronal *Cd2ap* deletion had no obvious influence on human Aβ level. *n* = 3 (APP/PS1, female *n* = 1, male *n* = 2), *n* = 5 (CKO × APP/PS1, female *n* = 3, male *n* = 2). **g** ELISA analysis of Aβ showed that neuronal *Cd2ap* deletion had no obvious influence on murine Aβ level. *n* = 6 (WT, female *n* = 2, male *n* = 4), *n* = 6 (APP/PS1, female *n* = 3, male *n* = 3), *n* = 4 (CKO, female *n* = 3, male *n* = 1), *n* = 6 (CKO × APP/PS1, female *n* = 4, male *n* = 2). **h-j** In 4.5-month-old mice, Immunoblots revealed that neuronal *Cd2ap* deletion had no obvious influence on the full-length APP (APP-FL) and APP-CTF proteins. *n* = 6 (WT, female *n* = 3, male *n* = 3), *n* = 6 (APP/PS1, female *n* = 3, male *n* = 3), *n* = 6 (CKO, female *n* = 3, male *n* = 3), *n* = 6 (CKO × APP/PS1, female *n* = 3, male *n* = 3). **k, l** In 4.5-month-old mice, Immunoblots revealed that neuronal *Cd2ap* deletion led to significantly increased ptau202/205 (AT8) and p-tau396 level, especially in the CKO × APP/PS1 mice. *n* = 7 (WT, female *n* = 3, male *n* = 4), *n* = 7 (APP/PS1, female *n* = 3, male *n* = 4), *n* = 7 (CKO, female *n* = 4, male *n* = 3), *n* = 7 (CKO × APP/PS1, female *n* = 4, male *n* = 3). All data are presented as mean ± SEM. Unpaired* t*-test with two-tailed analysis (**f)**, one-way ANOVA with Turkey’s multiple comparison tests for multiple comparisons (**b-e, g, i, j, k)**, Kruskal–Wallis tests with Dunn’s multiple comparison tests (**l)**. *P < 0.05, **P < 0.01, ***P < 0.001, ****P < 0.0001

This correction does not affect the description of the results or the conclusion of this work.

The original article [1] has been updated.
