# Supplementary material for: CD2AP deficiency aggravates Alzheimer’s disease phenotypes and pathology through p38 MAPK activation
Source: Transl Neurodegener. 2024 Dec 19;13:64. doi: 10.1186/s40035-024-00454-5 (PMC11657702; doi:10.1186/s40035-024-00454-5)
Supplement: Supplementary file 1 — Figure S1. Expression of CD2AP in different adult mouse tissues. Figure S2. The body weights of Cd2ap KO and CKO mice and pathological staining in CKO mice. Figure S3. No obvious difference in Aβ burden between APP/PS1 and CKO × APP/PS1 mice was observed. Figure S4. Neuronal Cd2ap deletion increase p-tau level in mice. Figure S5. Neuronal Cd2ap deletion did not influence the activation of microglia and astrocytes. Figure S6. Neuronal Cd2ap deletion exerted no influence in GSK-3β, CDK5, or p35 pathway. Table S1. Primer sequences for genotyping and qPCR. [file 40035_2024_454_MOESM1_ESM.docx]

**Supplementary** **Information for**

**CD2AP deficiency aggravates Alzheimer’s disease phenotypes and pathology through p38 MAPK activation**

Yan-Yan Xue^1#^; Zhe-Sheng Zhang^1#^; Rong-Rong Lin^1^; Hui-Fen Huang^1^; Ke-Qing Zhu^2^; Dian-Fu Chen^1,3^; Zhi-Ying Wu^1,3,4^**^*^**; Qing-Qing Tao^1*^

^1^Department of Neurology and Research Center of Neurology in Second Affiliated Hospital, and Liangzhu Laboratory, Zhejiang University School of Medicine, Hangzhou 310009, China

^2^National Health and Disease Human Brain Tissue Resource Center and Department of Pathology, School of Medicine, Zhejiang University, Hangzhou 310058, China

^3^MOE Frontier Science Center for Brain Science and Brain-Machine Integration, School of Brain Science and Brain Medicine, Zhejiang University, Hangzhou 310058, China

^4^CAS Center for Excellence in Brain Science and Intelligence Technology, Shanghai 200031, China

^#^These authors contributed equally to this work.

**^*^Corresponding authors**

Email: [qingqingtao@zju.edu.cn](mailto:qingqingtao@zju.edu.cn) (Qing-Qing Tao); zhiyingwu@zju.edu.cn (Zhi-Ying Wu)


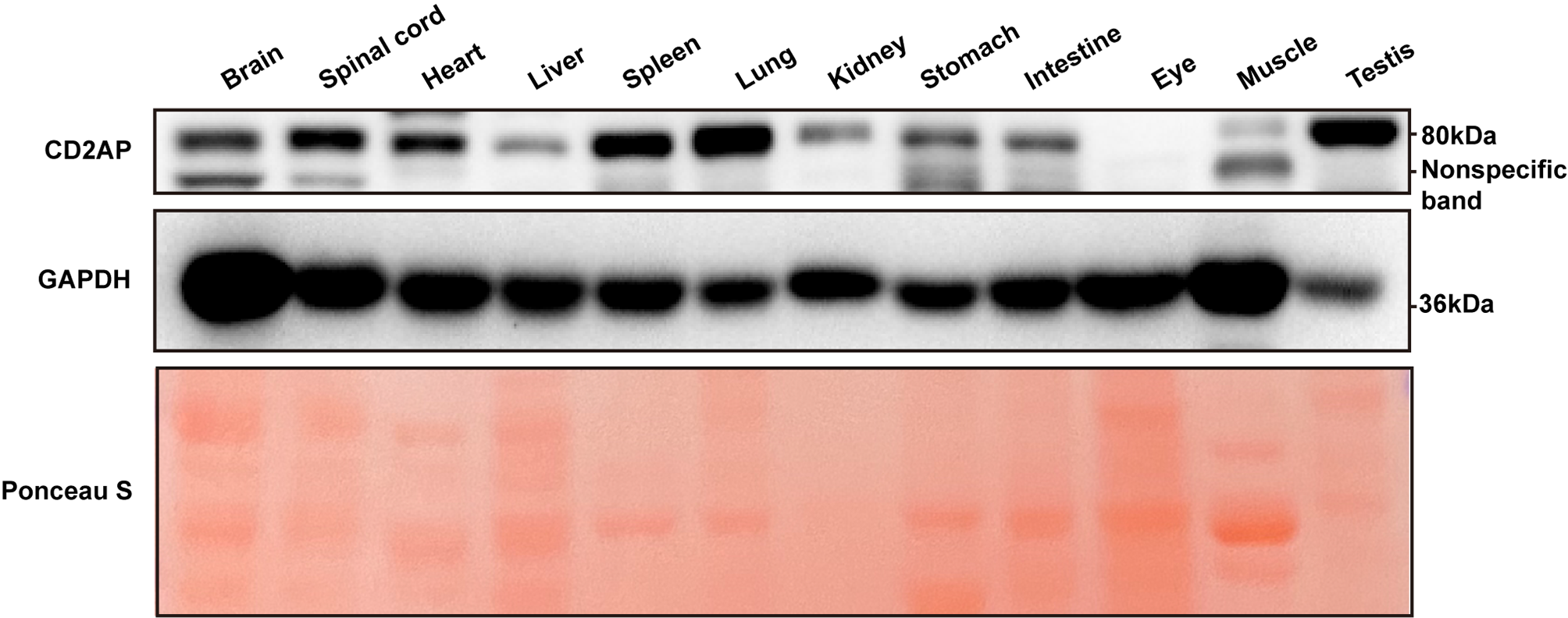


**Figure S1. Expression of CD2AP in different adult mouse tissues.** Immunoblots showing CD2AP protein level in different organs from 3-month-old mice.


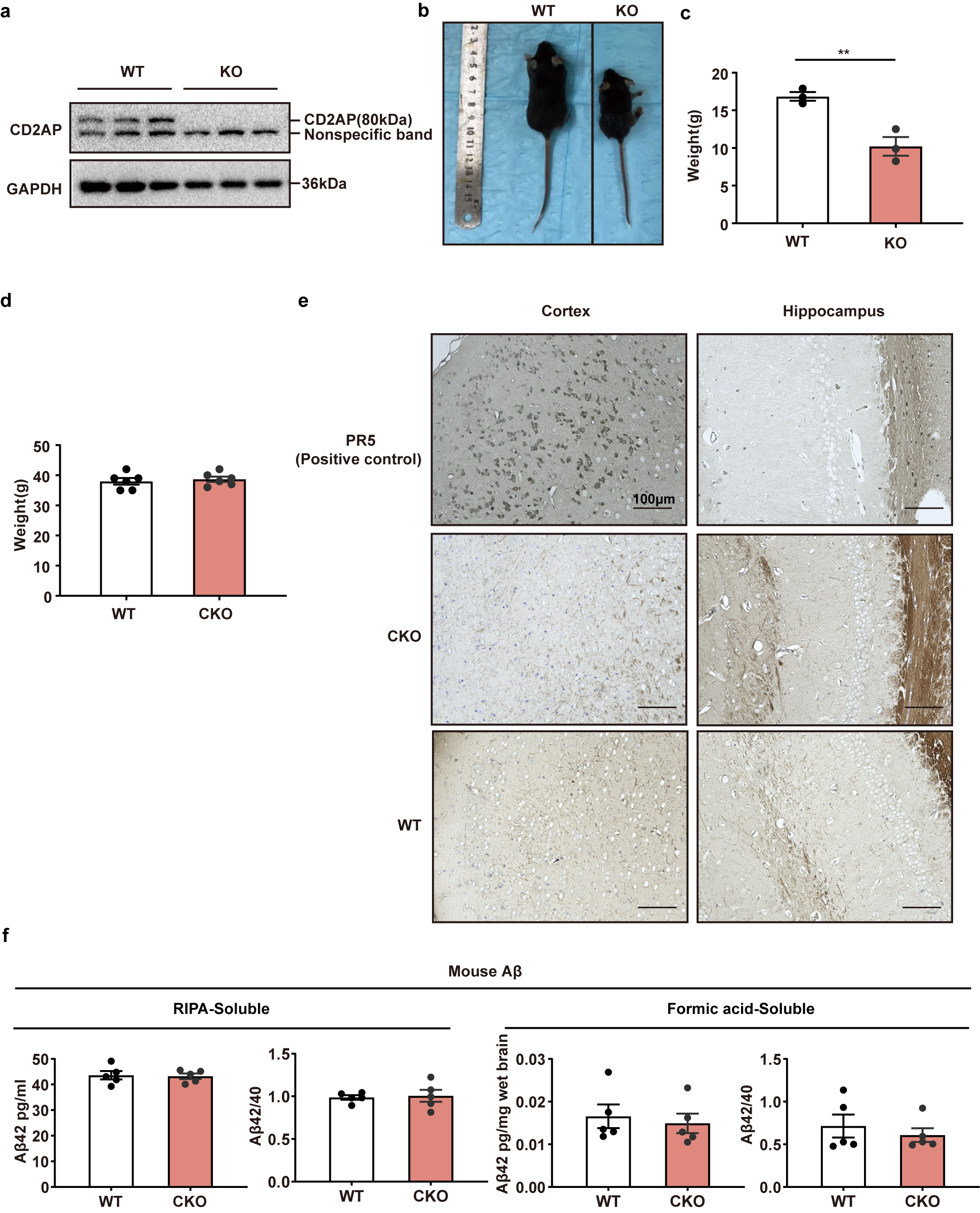


**Figure S2. The body weights of *Cd2ap* KO and CKO mice and pathological staining in CKO mice.** (a) Immunoblots showing CD2AP protein level in the 1-month-old mice brains after knockout of *Cd2ap*. (b,c) Representative photographs of WT (left) and *Cd2ap* KO (right) mice, and body weight statistics revealed that 1-month-old KO mice weighed significantly less than sex- and age-matched WT mice. (d) Body weights of 1-month-old CKO mice were not statistically different from sex- and age-matched WT mice. (e) Silver staining showed no obvious neurofibrillary tangles were observed in 15-month-old CKO mice, and the brain sections from 8-month-old PR5 mice were stained as a positive control and WT mice as a negative control. (f) ELISA analysis of murine Aβ showed that neuronal *Cd2ap* deletion had no obvious influence on murine Aβ level in 15-month-old mice. n=5 (WT), n=5 (CKO). All data are presented as mean ± SEM. Unpaired t-test with two-tailed analysis. **P* < 0.05, ***P* < 0.01.


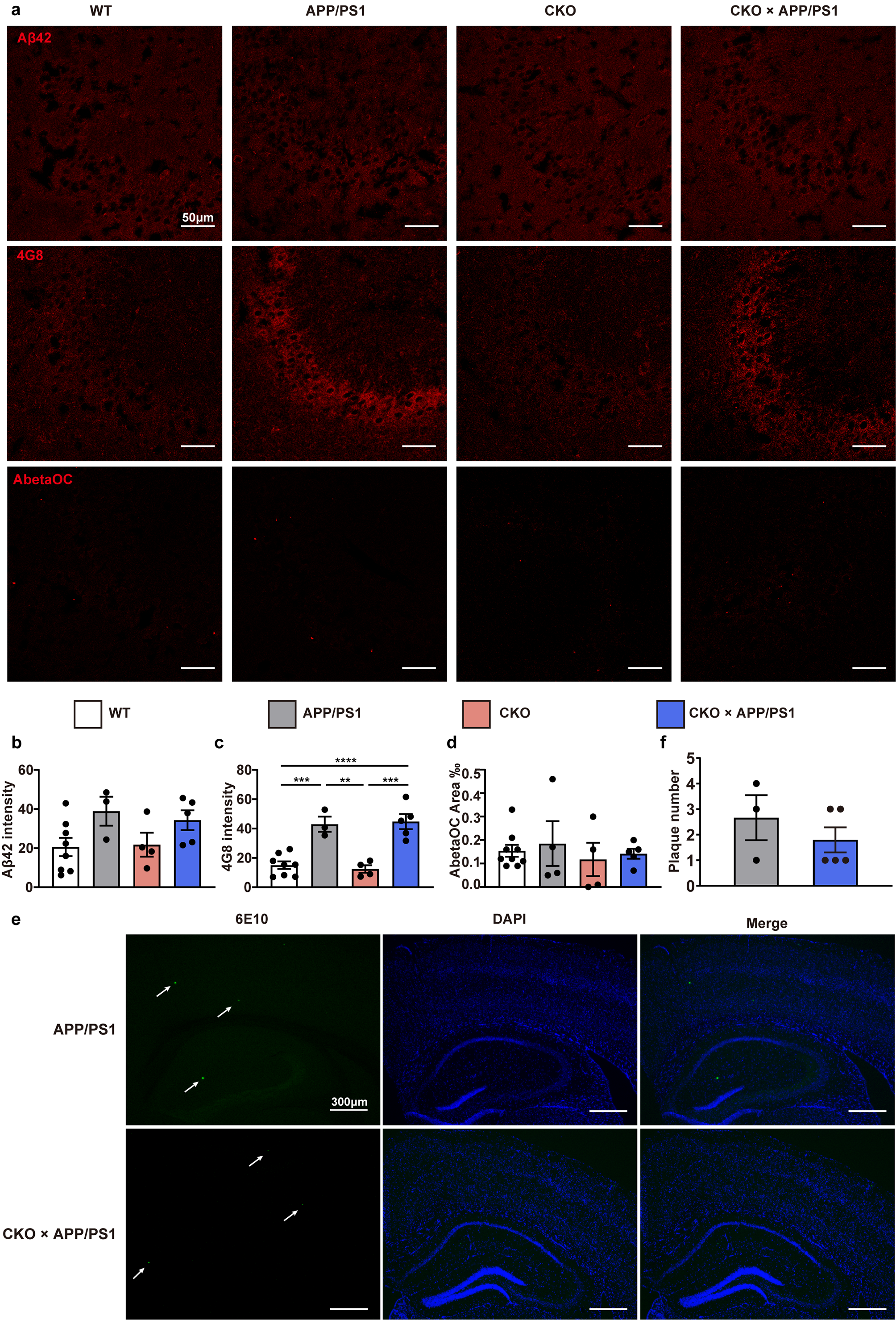


**Figure S3. No obvious difference in Aβ burden between APP/PS1 and CKO** × **APP/PS1 mice was observed.** (a) Representative images of the CA3 region in the hippocampus stained with anti-Aβ42, pan Aβ (4G8) and amyloid fibrils (AbetaOC) in 4.5-month-old mice. (b, c) Quantification of the intensity of Aβ42 and pan Aβ (4G8), n=8 (WT), n=3 (APP/PS1), n=4 (CKO), n=5 (CKO × APP/PS1). (d) Quantification of the AbetaOC area, n=9 (WT), n=4 (APP/PS1), n=4 (CKO), n=5 (CKO × APP/PS1). (e, f) Immunofluorescence analysis showed there was no significant difference in the plaque numbers in the brain of 4.5-month-old CKO × APP/PS1 mice compared with APP/PS1 mice, n=3 (APP/PS1), n=5 (CKO × APP/PS1). All data are presented as mean ± SEM. One-way ANOVA with Turkey’s multiple comparison tests for multiple comparisons (b, c), Kruskal-Wallis tests with Dunn’s multiple comparison tests (d), unpaired t-test with two-tailed analysis (f). **P* < 0.05, ***P* < 0.01, ****P* < 0.001, *****P* < 0.0001.


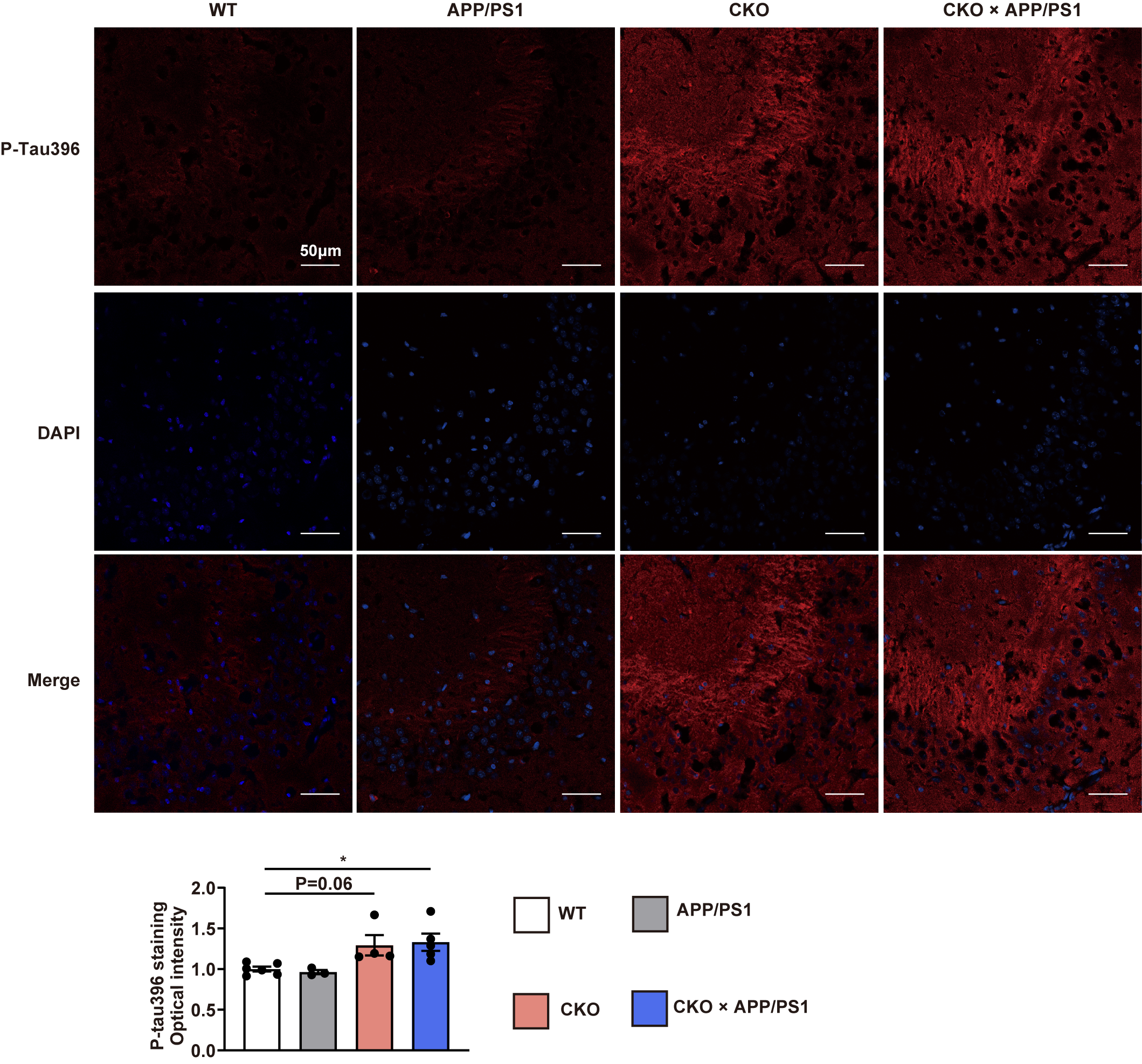


**Figure S4. Neuronal *Cd2ap* deletion increase p-tau level in mice.** Immunofluorescence analysis showed increased p-tau 396 level in 4.5-month-old CKO and CKO × APP/PS1 mice. Kruskal-Wallis tests with Dunn’s multiple comparison tests, **P* < 0.05.


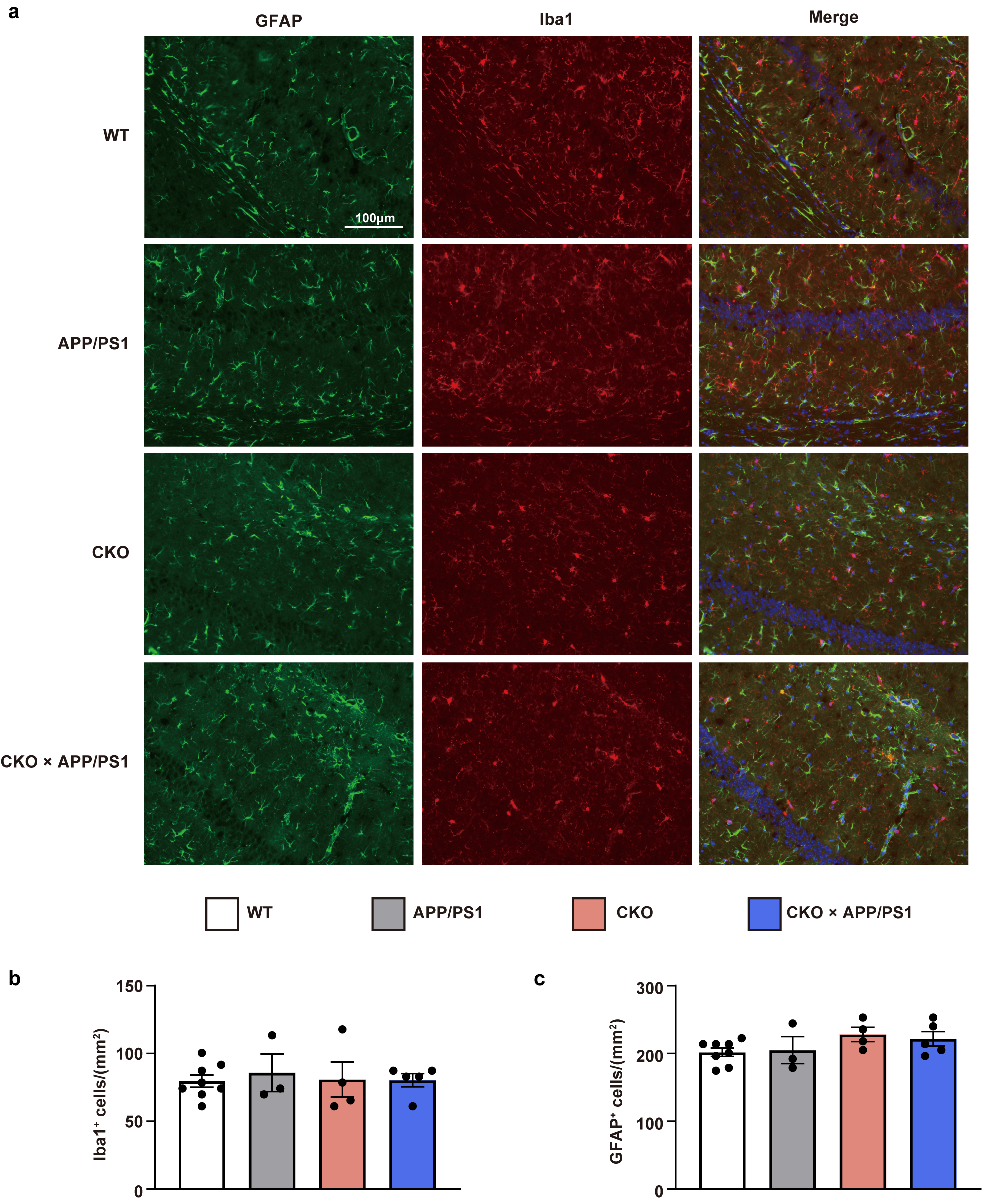


**Figure S5. Neuronal *Cd2ap* deletion did not influence the activation of microglia and astrocytes.** Immunofluorescence analysis indicated that the number of Iba1-positive and GFAP-positive cells showed no significant changes in both 4.5-month-old CKO and CKO × APP/PS1 mice. Kruskal-Wallis tests with Dunn’s multiple comparison tests (b), One-way ANOVA with Tukey’s multiple comparison tests for multiple comparisons (c).

**
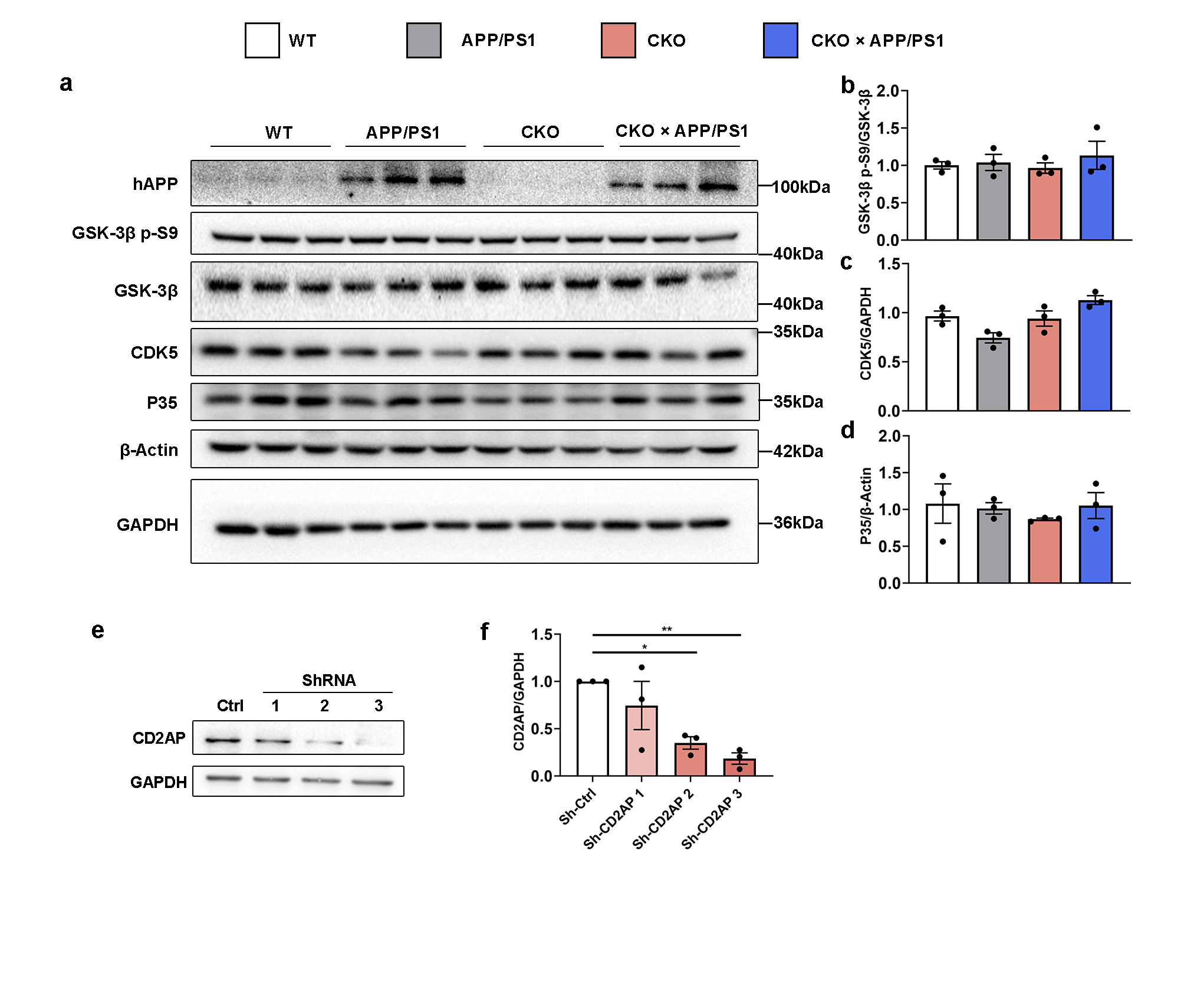
 Figure S6.** **Neuronal *Cd2ap* deletion exerted no influence in GSK-3β, CDK5, or p35 pathway.** (a-d) The deficiency of CD2AP exerted little influence on the levels of CDK5, p35, or phosphorylation of GSK-3β in 4.5-month-old mice. (e, f) CD2AP was obviously knocked down in SH-SY5Y cells using Sh-CD2AP 2 and Sh-CD2AP 3. All data are presented as mean ± SEM. One-way ANOVA with Dunnett’s multiple comparison tests for multiple comparisons. **P* < 0.05, ***P* < 0.01.

**Table S1. Primer sequences for genotyping and qPCR**

|  | Name | 5’-3’ sequence |
| --- | --- | --- |
| Genotyping primers | OIMR1597 | gACTgACCACTCgACCAggTTCTg |
|  | OIMR1598 | CTTgTAAgTTggATTCTCATATCCg |
|  | OIMR1644 | AATAGAGAACGGCAGGAGCA |
|  | OIMR1645 | GCCATGAGGGCACTAATCAT |
|  | 42 | CTAGGCCACAGAATTGAAAGATCT |
|  | 43 | GTAGGTGGAAATTCTAGCATCATCC |
|  | 5'loxp-Fw | gatttccaggagctgggatgatggg |
|  | 5'loxp-Rv | tcatcaggtaaggtgcttgcctacc |
|  | 3'loxp-Fw | aacaccagacacttccaacccttta |
|  | 3'loxp-Rv | ctcattgggcatcaacatgtcacact |
|  | Syn1-MSD-Fw | atcgGGATCCacattcgcctcagtctcagcttc |
|  | iCre-Mut-Rv | GCACACAGACAGGAGCATCTTC |
|  | Syn1-MSD-Fw | atcgGGATCCacattcgcctcagtctcagcttc |
|  | Syn1-MSD-Rv | atcgCTCGAGagagctccaggagaggattcgatg |
|  | Cre-Fw | CAGAACCTGAAGATGTTCGC |
|  | Cre-Rv | CCTGATCCTGGCAATTTCGG |
| QPCR primers | Mouse GAPDH Fw | GGTTGTCTCCTGCGACTTCA |
|  | Mouse GAPDH Rv | TGGTCCAGGGTTTCTTACTCC |
|  | Mouse cd2ap fw | CTCTCCACAAAATGAGGACGAA |
|  | Mouse cd2ap Rv | CTTATTGTTCAGGGTTCCACTCC |
