# Supplementary figures and images for: CD2AP deficiency aggravates Alzheimer’s disease phenotypes and pathology through p38 MAPK activation
Source: Transl Neurodegener. 2024 Dec 19;13:64. doi: 10.1186/s40035-024-00454-5 (PMC11657702; doi:10.1186/s40035-024-00454-5)

Uncropped full-length pictures of Western blotting membranes presented in the figures.


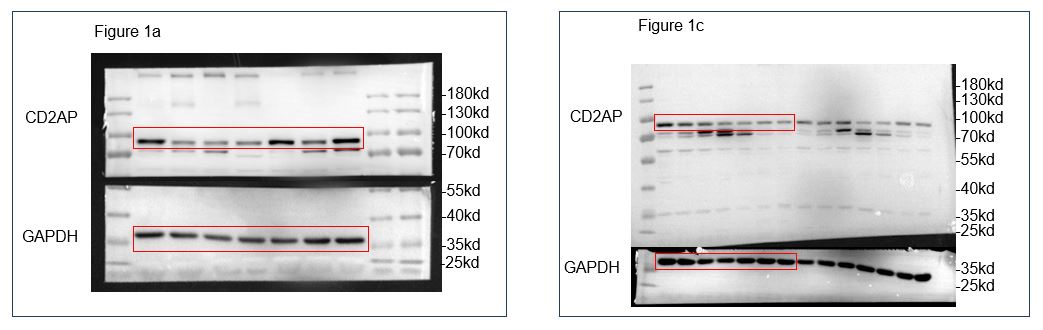

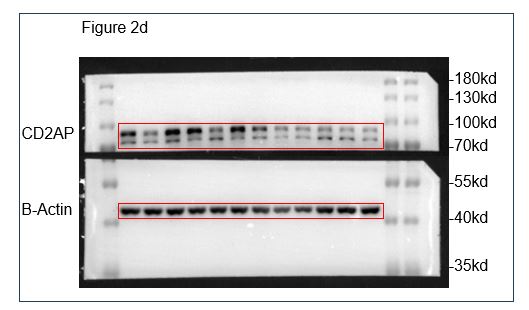


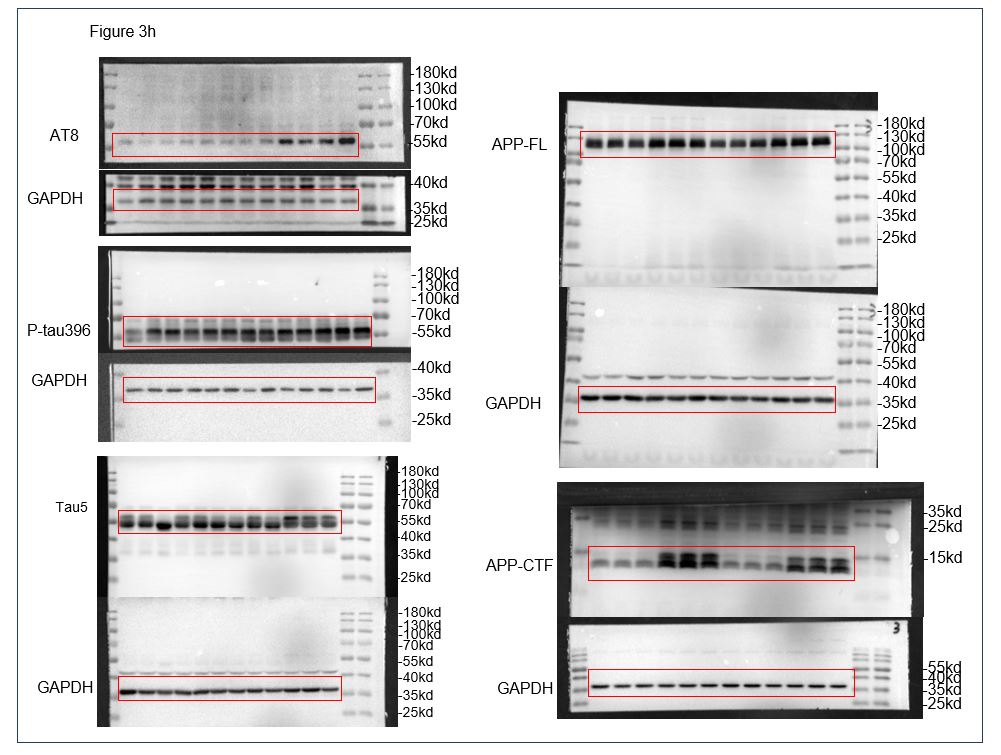

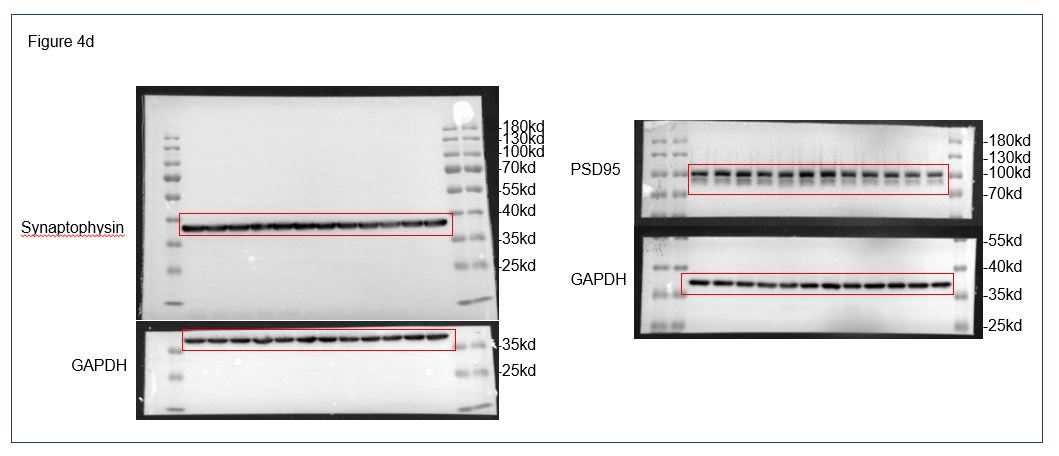

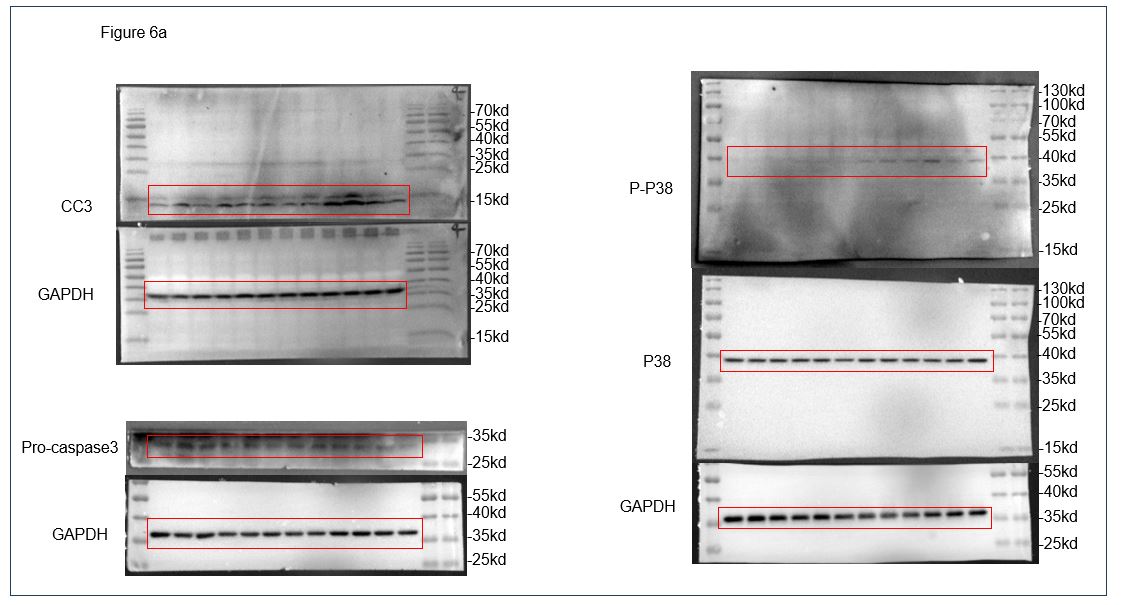

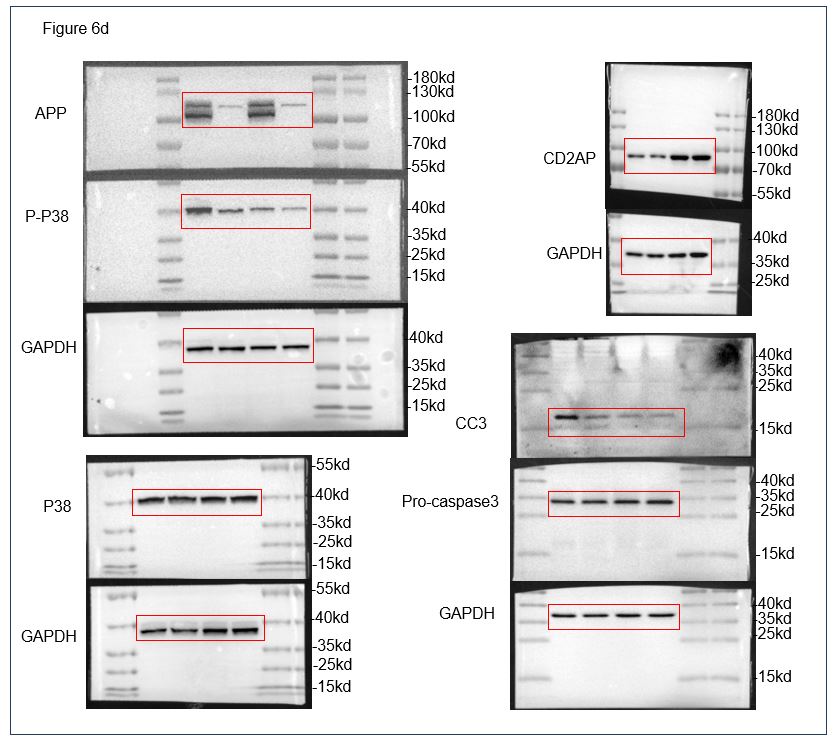

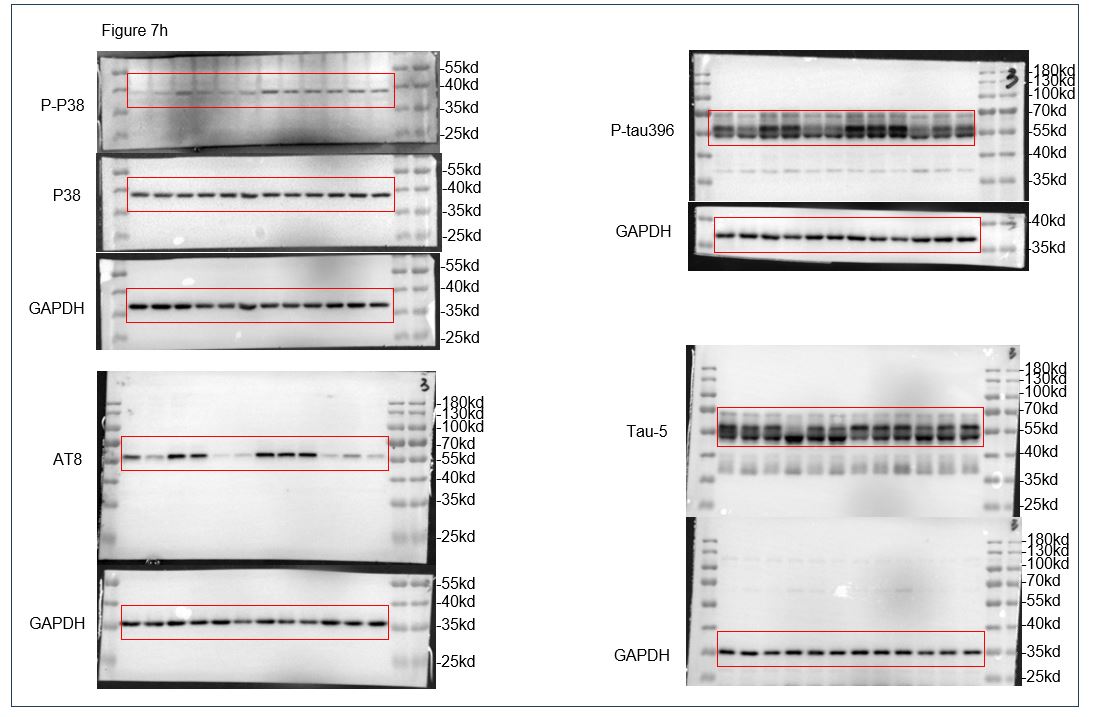

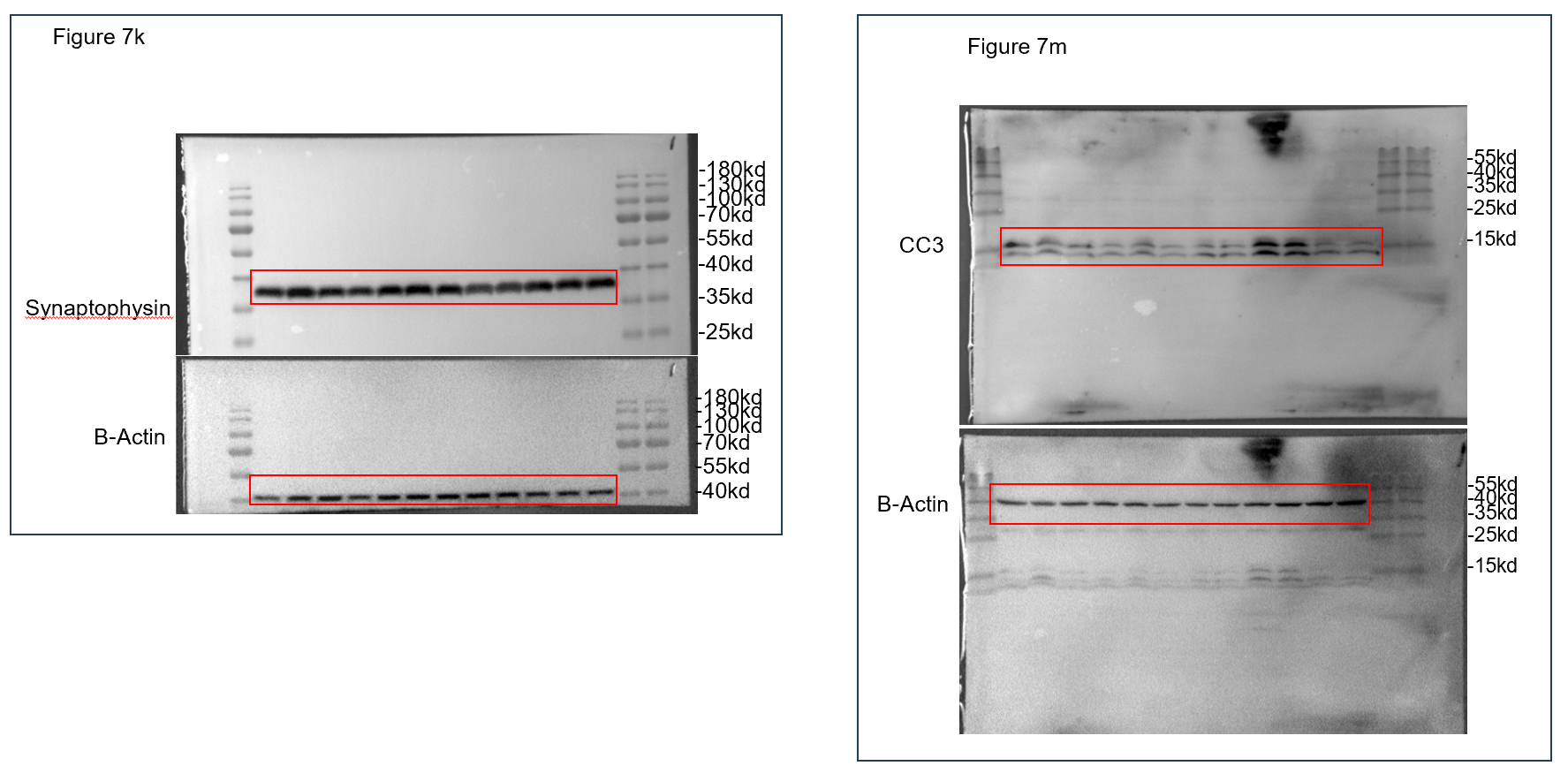

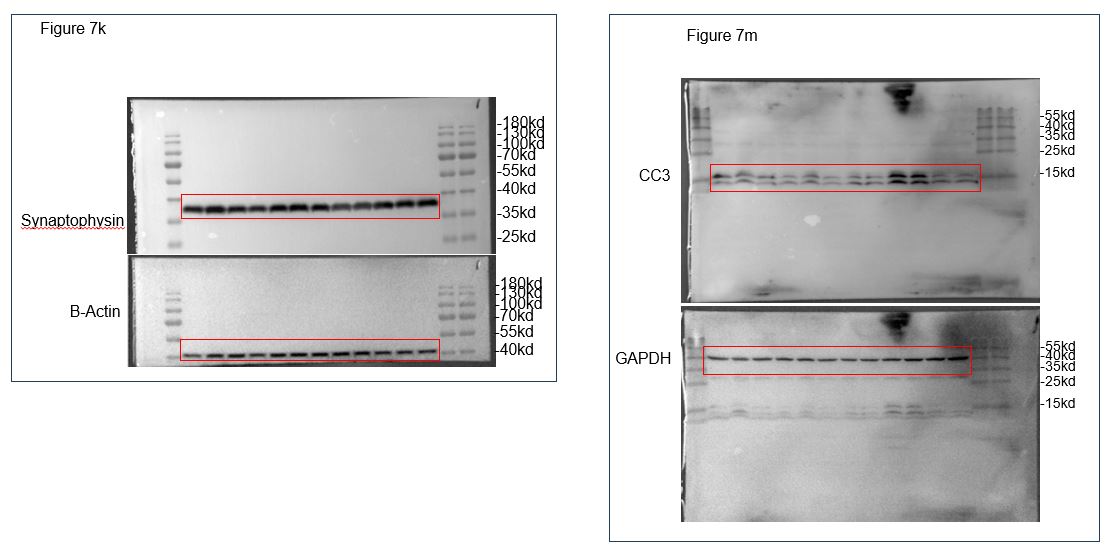

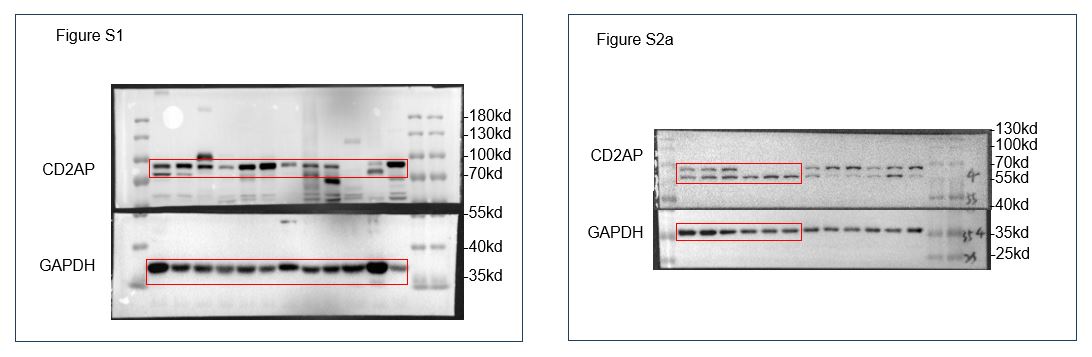

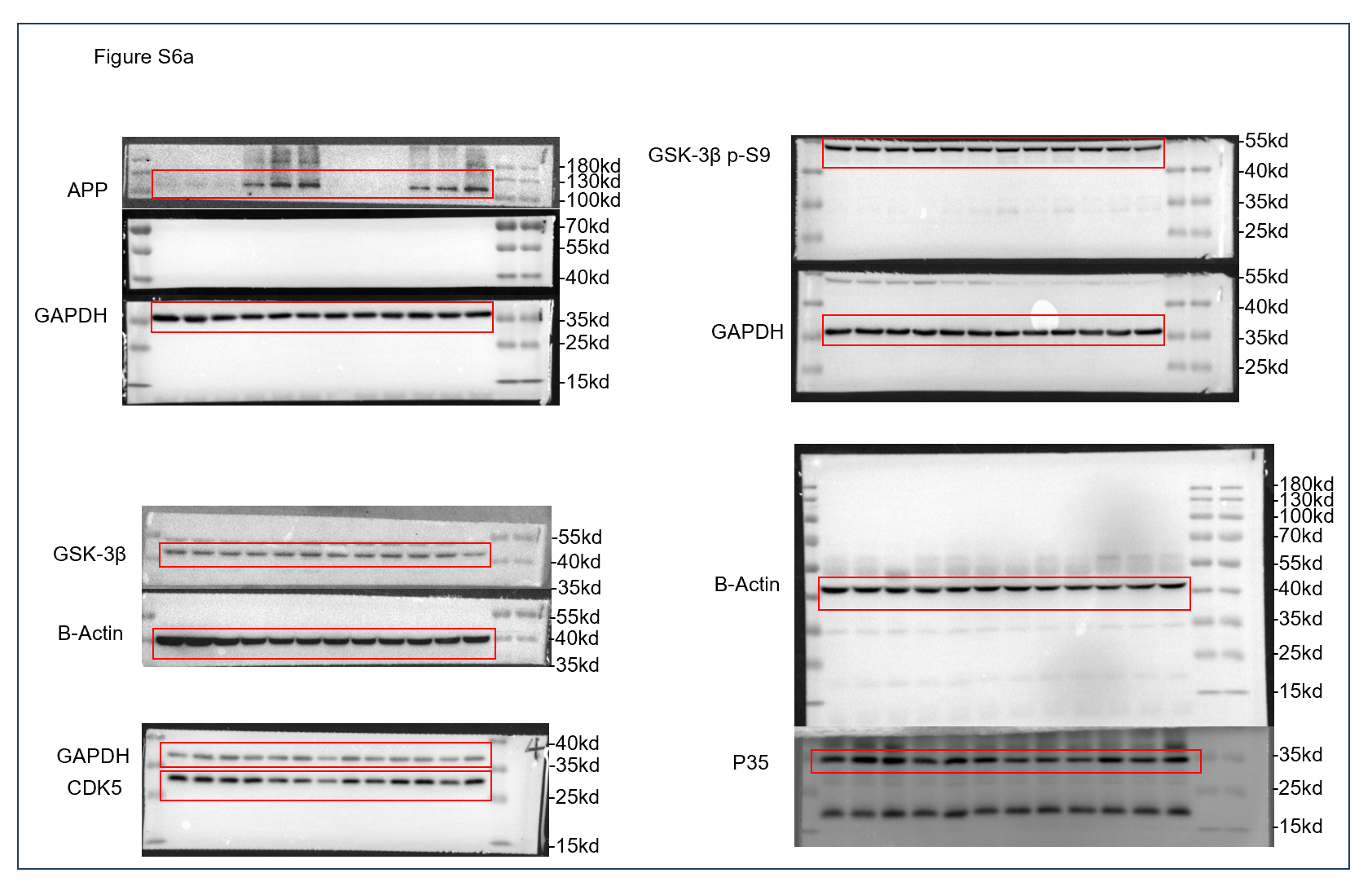

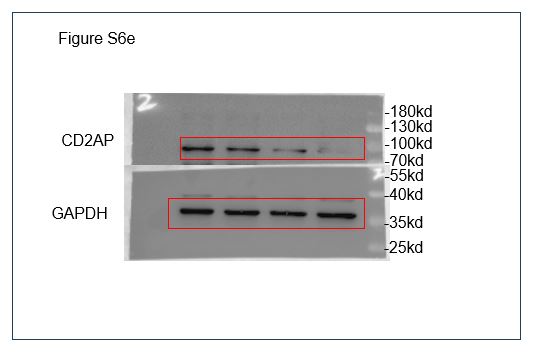

Supplement: Supplementary file 3 — Uncropped full-length pictures of Western blotting membranes presented in the figures. [file 40035_2024_454_MOESM3_ESM.docx]
